# A multifunctional “three-in-one” fluorescent theranostic system for hepatic ischemia–reperfusion injury†

**DOI:** 10.1039/d4sc04962d

**Published:** 2024-11-13

**Authors:** Jihong Liu, Dongni Yin, Wen Zhang, Xin Wang, Tony D. James, Ping Li, Bo Tang

**Affiliations:** a College of Chemistry, Chemical Engineering and Materials Science, Key Laboratory of Molecular and Nano Probes, Ministry of Education, Collaborative Innovation Center of Functionalized Probes for Chemical Imaging in Universities of Shandong, Institutes of Biomedical Sciences, Shandong Normal University China zhangwen@sdnu.edu.cn lip@sdnu.edu.cn tangb@sdnu.edu.cn; b College of Chemistry and Chemical Engineering, Northwest Normal University Lanzhou 730070 People's Republic of China; c Laoshan Laboratory Qingdao 266237 People's Republic of China; d Department of Chemistry, University of Bath Bath BA2 7AY UK t.d.james@bath.ac.uk; e Key Laboratory for Advanced Materials, Joint International Research Laboratory of Precision Chemistry and Molecular Engineering, Feringa Nobel Prize Scientist Joint Research Center, Shanghai Key Laboratory of Functional Materials Chemistry, Institute of Fine Chemicals, Frontiers Science Center for Materiobiology and Dynamic Chemistry, School of Chemistry and Molecular Engineering, East China University of Science and Technology Shanghai 200237 People's Republic of China; f School of Chemistry and Chemical Engineering, Henan Normal University Xinxiang 453007 People's Republic of China

## Abstract

Hepatic ischemia–reperfusion injury (HIRI) is the main cause of postoperative liver dysfunction and liver failure. Traditional separation of HIRI diagnosis and therapy confers several disadvantages, including the inability to visualize the therapeutic and asynchronous action. However, developing a versatile material with integrated diagnosis and treatment for HIRI remains a great challenge. Given that hypochlorous acid (HOCl) plays a crucial oxidative role in HIRI, we developed a single-component multifunctional fluorescent theranostic platform (MB-Gly) with a “three-in-one” molecular design incorporating a near-infrared fluorophore methylene blue, glycine and a HOCl-response unit, which could not only provide real-time visualization of HIRI but also boost targeted drug delivery. Using MB-Gly, we were able to achieve real-time and dynamic monitoring of HOCl during HIRI in hepatocytes and mouse livers and reduce the liver damage in hepatocytes and mice. RNA sequencing illustrated the therapeutic role of MB-Gly associated with changes in gene expression related to apoptosis, oxidative stress, metabolism and inflammation. To the best of our knowledge, this is the first multifunctional fluorescent theranostic system for HIRI reported to date. Our smart “three-in-one” approach shines light on the etiology and pathogenesis of HIRI, providing profound insights into the development of potential therapeutic targets.

## Abstract 2

A single-component multifunctional fluorescent theranostic system (MB-Gly) for use during hepatic ischemia-reperfusion injury (HIRI) has been developed.

## Graphical abstract

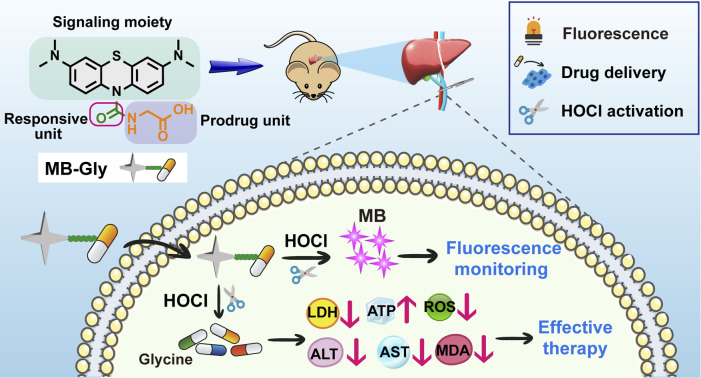

## Introduction

Hepatic ischemia–reperfusion injury (HIRI) is a pathological process involving prolonged ischemic injury of liver tissues and subsequent blood perfusion, which is responsible for liver dysfunction and liver failure after surgical operations such as liver resection and liver transplantation.^1^ HIRI not only induces the incidence of acute and chronic rejection^2^ but also causes secondary dysfunction of remote organs,^3^ which collectively affects the surgical outcome and patient prognosis. Early diagnosis and timely intervention provide the opportunity for alleviating or reversing the progression of HIRI but remain challenging. Previously, blood tests, imaging diagnosis and liver biopsy have been used for the main clinical diagnoses of HIRI.^4^ However, these conventional methods exhibit low sensitivity, poor spatiotemporal resolution, restricted signal penetration, and hysteresis and are invasive, thereby making them unable to offer real-time, non-invasive and dynamic visualization of HIRI progression.^5^ Meanwhile, surgical operation and pharmacological intervention have been used for the treatment of HIRI.^6^ Nonetheless, surgical treatments such as ischemic preconditioning, ischemic post-treatment and machine reperfusion are difficult to perform.^7^ Pharmacological intervention also faces certain drawbacks, for instance, non-specific biological distribution, poor pharmacokinetics and significant side effects after systemic administration.^8^ More importantly, the separate diagnosis and treatment of HIRI is unable to visualize liver lesions during treatment, release drugs in a controlled manner and enable simultaneous detection and realization of therapeutic action. Therefore, the development of a single-component integrated platform to achieve the synchronous early diagnosis and targeted treatment of HIRI is essential.

The occurrence of HIRI is intimately coupled with oxidative stress.^9^ Accumulating evidence indicates that high levels of hypochlorous acid (HOCl) (up to the millimolar concentration range) accumulate in inflammed lesions when ischemia–reperfusion injury occurs,^10^ whereas the normal physiological concentrations of HOCl in living organisms are typically in the range of 5–25 μM.^11^ The major oxidant in HIRI, HOCl, destroys intracellular protein, lipids and DNA during HIRI, leading to hepatocyte death and tissue dysfunction.^12^ Excessive HOCl also induces mitochondrial dysfunction, calcium ion (Ca^2+^) overload, and mitochondrial permeability transition, and accelerates cell death.^13^ As such, HOCl could act as an early biomarker for accurate diagnosis of HIRI and might also function as a key target for effective remission of HIRI. As a potential hepatoprotective drug, glycine has attracted significant interest in the treatment of HIRI due to high antioxidant, anti-inflammatory and immunomodulatory activities.^14^ In particular, glycine plays multiple protective roles during HIRI by preventing intracellular Ca^2+^ overload, causing chloride ion influx, inhibiting protease activity and inflammatory cytokine production, as well as reducing lipid peroxidation.^15–17^ However, the therapeutic effect of glycine is significantly hampered by non-specific biological distribution and insufficient liver uptake upon systemic administration in complicated biological environments.^18^ Hence, the development of a diagnostic and therapeutic tool that can release glycine on demand triggered by excessive HOCl during HIRI is highly desirable.

Fluorescence imaging exhibits high sensitivity, outstanding selectivity, excellent temporal–spatial resolution, simple operation and rapid response, which provides hope for monitoring biological events in living systems and the diagnosis of diseases.^19–24^ The past few years have witnessed the rapid development of small-molecule fluorescent probes and theranostic prodrugs for HIRI detection and therapy.^25–29^ Unfortunately, no integrated diagnosis and treatment system for HIRI has been developed to date. Reactive oxygen species (ROS)-responsive therapeutic prodrugs represent an appropriate tool for the early diagnosis and targeted treatment of HIRI in highly dynamic and complicated environments, providing fluorescence monitoring and localized drug release.^30^ Diseases associated with redox imbalance and the accumulation of ROS have resulted in the development of ROS-triggered activation strategies for the construction of fluorescent theranostic prodrugs.^31,32^ The integration of a fluorophore, drug and ROS-responsive unit into one compound enables early diagnosis, targeted drug delivery and real-time visualization, providing strategies for the simultaneous detection and treatment of diseases.^32–36^

**Scheme 1:**
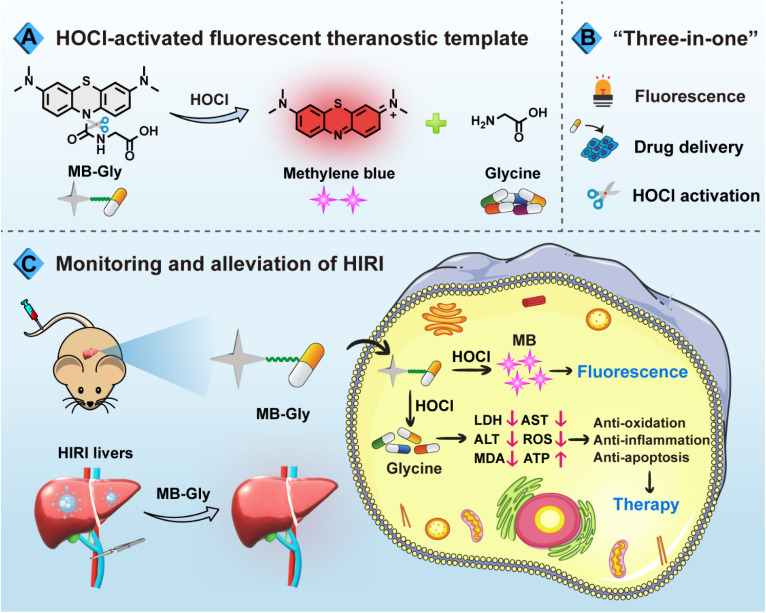
Schematic diagram of the fluorescence regulation mechanism and the action of MB-Gly during HIRI. (A) Luminescence mechanism of MB-Gly and the release of MB and glycine. (B) “Three-in-one” strategy of MB-Gly integrated with NIR fluorescence signaling, drug delivery and HOCl activation. (C) Multifunctional fluorescent theranostic material (MB-Gly) for monitoring and alleviation of HIRI mice.

Based on the need for a synergistic diagnosis and treatment system for HIRI, we have developed a single-component multifunctional fluorescent theranostic system (MB-Gly) (Scheme 1). MB-Gly contains a near-infrared (NIR) fluorophore methylene blue (MB), glycine and a HOCl-triggered masking group. As such, the *in situ* biomarker HOCl could trigger MB-Gly to simultaneously release the NIR fluorophore MB and glycine during HIRI. *In vitro* and *in vivo* experiments indicated that MB-Gly was endowed with a NIR fluorescence signal response and glycine delivery capability due to HOCl-triggered activation. MB-Gly achieved real-time visualization of HOCl fluctuations in HIRI processes. Furthermore, the therapeutic efficacy of a series of potential drugs for HIRI was comprehensively evaluated. Notably, multiple biomarker assessments and RNA sequencing confirmed the versatility of MB-Gly for the alleviation of liver injury, which involved anti-oxidation, anti-inflammation and anti-apoptosis. The smart “three-in-one” strategy provides promise for the accurate diagnosis and targeted therapy of HIRI. To the best of our knowledge, this is the first multifunctional HOCl-driven theranostic agent for HIRI, paving the way for the development of therapeutic targets and evaluation of key signaling pathways.

## Results and discussion

### Design and synthesis of MB-Gly

**Fig. 1:**
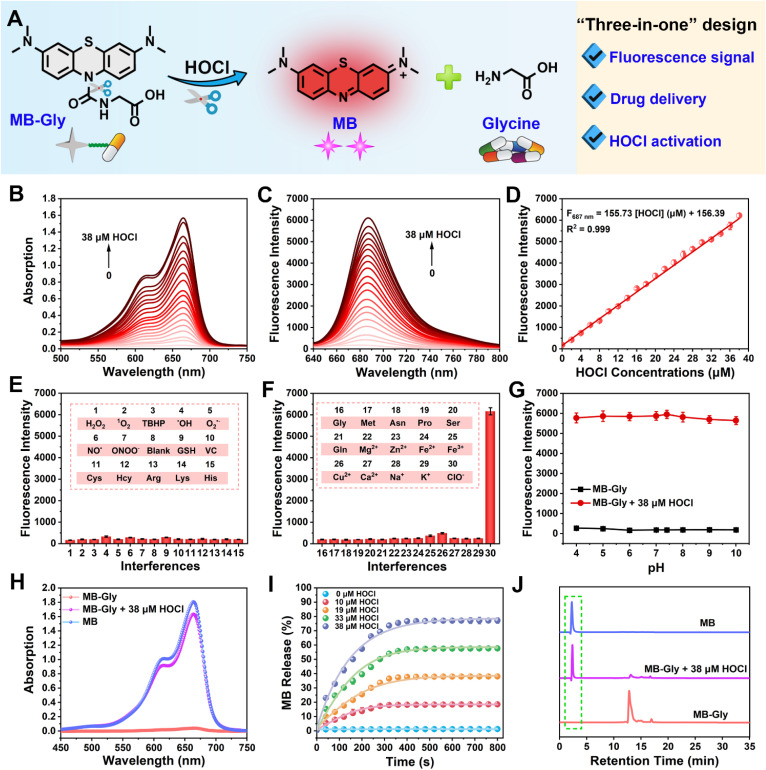
Spectral response of MB-Gly toward HOCl. (A) Schematic diagram of MB and glycine released by MB-Gly in response to HOCl. (B) Absorption spectra of MB-Gly (25 μM) after reaction with HOCl (0–38 μM). (C) Fluorescence spectra of MB-Gly after reaction with HOCl (0–38 μM). *λ*_ex_ = 620 nm. (D) Linear relationship of the fluorescence intensity of MB-Gly at 687 nm with HOCl. (E) and (F) Fluorescence response of MB-Gly to ROS, RNS, amino acids and metal ions. *λ*_ex/em_ = 620/687 nm. (G) Fluorescence intensity changes of MB-Gly before and after reaction with HOCl in different pH environments. (H) Absorption spectra of MB, MB-Gly before and after the reaction with HOCl (38 μM). (I) Release efficiency of MB from MB-Gly in the presence of HOCl. (J) HPLC analysis of MB, MB-Gly and MB-Gly after reaction with HOCl.

As an FDA-approved photosensitizer and high-performance fluorophore, MB is widely used in biomedical research and for clinical diagnosis due to its NIR fluorescence emission, low background interference and good biocompatibility.^37,38^ In particular, MB plays antioxidant and anti-inflammatory roles in HIRI by stabilizing energy metabolism, inhibiting lipid peroxidation and regulating pro-inflammatory factor signaling pathways.^39^ Meanwhile, glycine exhibits significant protective effects on cell death induced by HIRI. For instance, glycine activates glycine receptors, inhibiting the activation of Kupffer cells and the release of cytokines.^40^ Moreover, glycine can inactivate calcium-dependent proteolytic enzymes and reduce the damage of membrane lipid peroxidation caused by ROS.^16^ Consequently, using MB as a NIR fluorophore in combination with glycine as an active drug coupled using a urea reactive linker, we developed MB-Gly as an integrated diagnosis and treatment platform (Scheme 1A, B and Fig. 1A). Upon activation by HOCl, the urea bond in MB-Gly was cleaved,^41,42^ resulting in the release of MB and glycine. This generated a fluorescence response and caused the effective release of glycine, enabling effective NIR fluorescence-driven targeted therapy, which significantly reduced ROS production, inflammatory response and cell apoptosis (Scheme 1C). The MS and NMR characterization results of MB-Gly are provided in Fig. S10–S12.†

### Optical properties of MB-Gly toward HOCl

The absorption spectra of MB-Gly stimulated by different concentrations of HOCl in PBS buffer solution (10 mM, pH = 7.4) were recorded. As shown in Fig. 1B, the absorption peak of MB-Gly was negligible at 664 nm, and the intensity gradually increased with the addition of HOCl. Accordingly, the fluorescence emission of MB-Gly at 687 nm was significantly enhanced after incubation with HOCl (0–38 μM) (Fig. 1C). Notably, 38 μM HOCl triggered a 33-fold fluorescence enhancement of MB-Gly at 687 nm, further demonstrating the effective release of the MB fluorophore. The fluorescence intensity of MB-Gly at 687 nm *versus* HOCl concentration (0–38 μM) was plotted and shown in Fig. 1D, which exhibits a good linear relationship with *F*_687 nm_ = 155.73 [HOCl] (μM) + 156.39, and the detection limit of MB-Gly toward HOCl was calculated to be 61 nM. These results confirmed that MB-Gly could respond to HOCl changes with high sensitivity, rendering the system suitable for detecting HOCl in living systems.

The specificity of MB-Gly toward HOCl was comprehensively evaluated. As shown in Fig. 1E and F, only HOCl induced significant enhancement of the fluorescence at 687 nm without interference from other ROS, reactive nitrogen species (RNS), metal ions and amino acids. However, the fluorescence response of MB-Gly toward HOCl was hardly affected by the coexistence of other biological interferents (Fig. S1†), illustrating the excellent selectivity of MB-Gly in response to HOCl. The fluorescence enhancement of MB-Gly upon HOCl addition was stable over a pH range from 4 to 10, indicating good pH stability (Fig. 1G). The photostability of MB-Gly was also evaluated. Upon the addition of HOCl, the red fluorescence of MB-Gly at 687 nm increased rapidly and reached a plateau within 7 min (Fig. S2†). As such, these *in vitro* experiments verified that MB-Gly exhibited high sensitivity, selectivity, resistance to pH interference and excellent photostability toward HOCl, ensuring the suitability of MB-Gly for monitoring HOCl under complex physiological conditions.

### Release of MB and glycine upon HOCl activation

Subsequently, the release of the MB fluorophore and glycine drug after the reaction of MB-Gly with HOCl was monitored using UV-vis spectroscopy. As shown in Fig. 1H, the characteristic absorption peak at 664 nm of MB-Gly emerged after adding 38 μM HOCl, which was consistent with the absorption of MB. The absorbance of MB at 664 nm exhibits a good linear relationship with the concentration of MB (Fig. S3†), so the release efficiencies of MB from MB-Gly at different concentrations of HOCl could be assessed. As shown in Fig. 1I, there was almost no generation of MB from MB-Gly in the absence of HOCl. The release efficiency of MB reached the maximum (*ca.* 77%) and remained stable for 400 s under the activation of 38 μM HOCl, indicating the effective conversion of MB-Gly to MB. Meanwhile, high performance liquid chromatography (HPLC) was used to characterize the production of MB from the reaction between MB-Gly and HOCl. As shown in Fig. 1J, MB-Gly and MB have well-separated retention times of 12.8 min and 2.3 min, respectively. After incubation with 38 μM HOCl, the HPLC peak of MB-Gly at 12.8 min decreased, while the corresponding peak at 2.3 min appeared, further confirming the consumption of MB-Gly and the production of MB. In addition, amino acid analysis by the HPLC system was used to identify the release of glycine in the HOCl-induced cleavage reaction of MB-Gly. In a solution containing MB-Gly and HOCl, a retention time of 8.3 min assigned to glycine was detected, verifying the existence of glycine after the reaction between MB-Gly and HOCl (Fig. S4†).^43^ Taken together, the above results confirmed the generation of the cleavage products MB and glycine from MB-Gly upon activation by HOCl.

### Fluorescence imaging of endogenous HOCl in hepatocytes

**Fig. 2:**
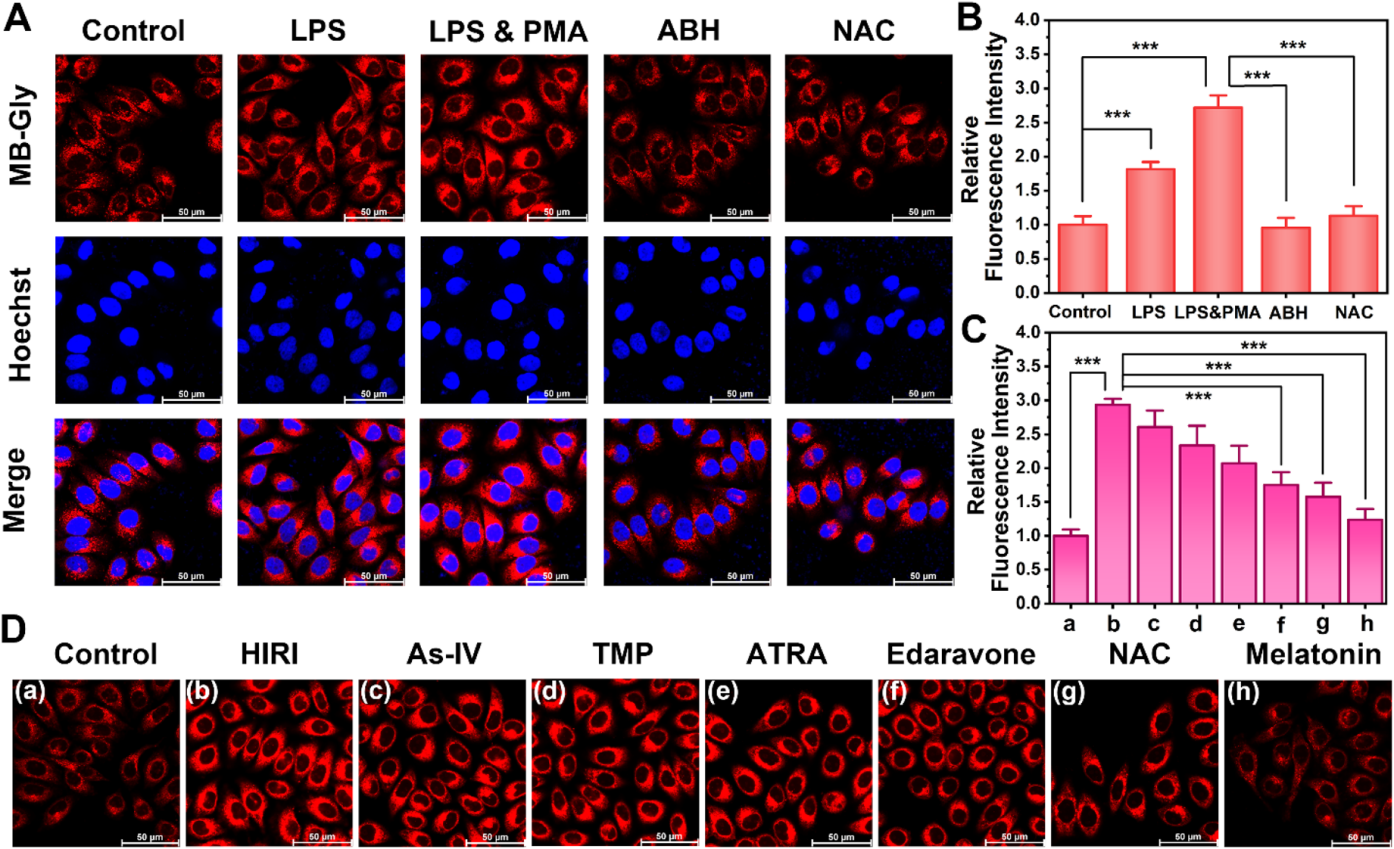
Fluorescence imaging analysis of endogenous HOCl in hepatocytes. (A) Fluorescence images of endogenous HOCl in hepatocytes treated with LPS (1 μg mL^−1^), LPS (1 μg mL^−1^) & PMA (1 μg mL^−1^), ABH (500 μM), and NAC (500 μM) by MB-Gly (50 μM, *λ*_ex_ = 638 nm, *λ*_em_ = 643–775 nm) and Hoechst 33342 (1 μg mL^−1^, *λ*_ex_ = 405 nm, *λ*_em_ = 420–535 nm). (B) and (C) Relative red fluorescence intensity output of (A) and (D), respectively. The red fluorescence intensity of the control group was defined as 1. (D) Fluorescence images of endogenous HOCl in the control group hepatocytes, HIRI group hepatocytes, and HIRI group pretreated with As-IV (500 μM), TMP (500 μM), ATRA (500 μM), edaravone (500 μM), NAC (500 μM), and melatonin (500 μM) by MB-Gly (50 μM, *λ*_ex_ = 638 nm, *λ*_em_ = 643–775 nm). The data are expressed as the mean ± SD. ***P < 0.001.

**Fig. 3:**
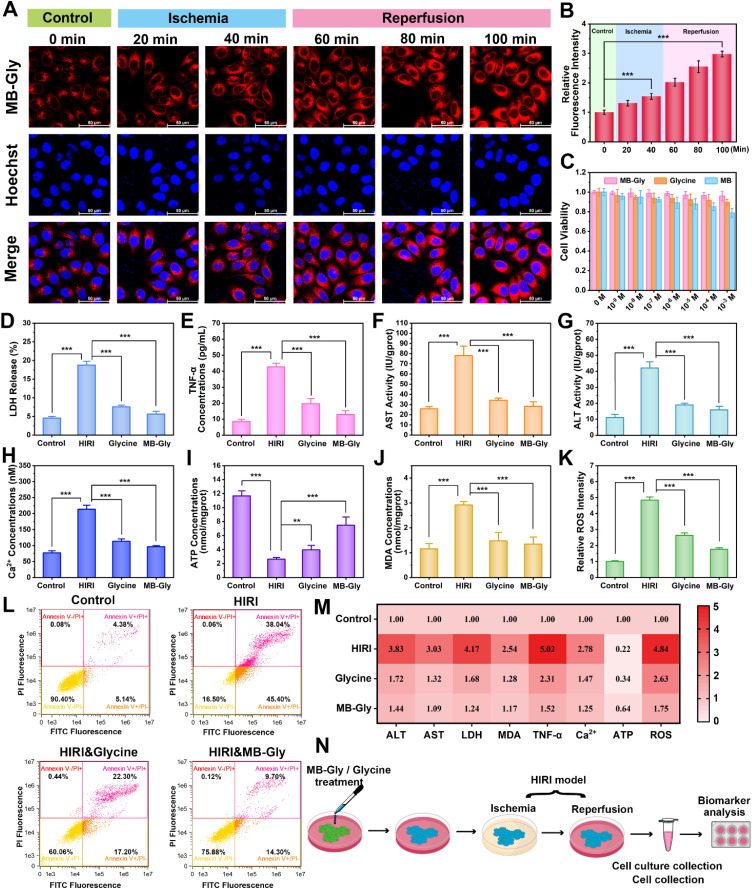
Fluorescence imaging analysis and therapeutic effect of MB-Gly on HIRI hepatocytes. (A) Fluorescence imaging of endogenous HOCl in hepatocytes during HIRI by MB-Gly (50 μM, *λ*_ex_ = 638 nm, *λ*_em_ = 643–775 nm) and Hoechst 33342 (1 μg mL^−1^, *λ*_ex_ = 405 nm, *λ*_em_ = 420–535 nm). (B) Relative red fluorescence intensity output of (A). The red fluorescence intensity of the control group was defined as 1. (C) Cytotoxicity assessment of MB-Gly, MB and glycine. (D) LDH release. (E) TNF-α release. (F) AST activity. (G) ALT activity. (H) Ca^2+^ content. (I) ATP content. (J) MDA content. (K) ROS levels. (L) Flow cytometry of hepatocytes. (M) Integration of multiple biomarkers in the control group, HIRI group, glycine-treated HIRI group and MB-Gly-treated HIRI group. The concentrations of all biomarkers in the control group were defined as 1. (N) Schematic diagram of assessment of the therapeutic effect of MB-Gly and glycine on HIRI hepatocytes.

Good biocompatibility is a prerequisite for the application of multifunctional fluorescent theranostic materials *in vivo*. The biological toxicity of MB-Gly and its cleaved products MB and glycine was comprehensively investigated in HL-7702 cells by CCK-8 assay. As shown in Fig. 3C, at different concentrations of MB-Gly, MB and glycine (0, 10^−9^, 10^−8^, 10^−7^, 10^−6^, 10^−5^, 10^−4^ and 10^−3^ M, respectively), the survival rates of hepatocytes were above 78%, which suggested good biocompatibility and negligible cytotoxicity of MB-Gly, MB and glycine. The capability of MB-Gly to image endogenous HOCl fluctuations in hepatocytes was then investigated using high-resolution confocal fluorescence microscopy. Lipopolysaccharide (LPS) and phorbol 12-myristate 13-acetate (PMA) were used as external stimuli to induce the production of endogenous HOCl in hepatocytes.^44^ Initially, the hepatocytes in the control group exhibited minimal fluorescence with MB-Gly (Fig. 2A), indicating low intracellular HOCl levels in the normal group. Then, upon treatment with 1 μg mL^−1^ LPS, HOCl-associated red fluorescence in the hepatocytes was significantly enhanced. In particular, stimulation with 1 μg mL^−1^ LPS and 1 μg mL^−1^ PMA resulted in a 2.7-times red fluorescence enhancement compared with the control group (Fig. 2B). In addition, a myeloperoxidase (MPO) inhibitor 4-aminobenzoic acid hydrazide (ABH) was used to inhibit the production of intracellular HOCl.^37^ As shown in Fig. 2A, the fluorescence of hepatocytes activated with LPS and PMA was effectively quenched upon ABH pretreatment. Similarly, an antioxidant *N*-acetylcysteine (NAC) could remove intracellular HOCl upon incubation with LPS and PMA.^44^ The red fluorescence intensity of NAC-pretreated hepatocytes was 0.41 times lower than that of LPS and PMA treated hepatocytes. These results indicated that MB-Gly enabled real-time and *in situ* visualization of endogenous HOCl levels in living cells.

### Real-time visualization of HOCl during HIRI and evaluation of the efficacy of HIRI drugs

After demonstrating the capacity of MB-Gly to image endogenous HOCl levels in hepatocytes, we explored the temporal and spatial dynamic behavior of HOCl in hepatocytes undergoing HIRI using MB-Gly. A HIRI model was established in hepatocytes by serum oxygen–glucose deprivation and subsequent reperfusion.^45^ To monitor HOCl levels during each phase of the HIRI process in real-time, the hepatocytes were first divided into six groups: control group, ischemia for 20 min group, ischemia for 40 min group, ischemia for 40 min and reperfusion for 20 min group, ischemia for 40 min and reperfusion for 40 min group, and ischemia for 40 min and reperfusion for 60 min group. As such, six groups of hepatocytes were incubated with MB-Gly for one-photon fluorescence imaging. As shown in Fig. 3A, in sharp contrast to the normal cells, the red fluorescence in HIRI hepatocytes increased gradually with the prolongation of ischemia. Interestingly, an enhanced HOCl-related red fluorescence was observed upon reperfusion, which reached a maximum intensity after 40 min of ischemia and 60 min of reperfusion (Fig. 3B), suggesting that HOCl could act as an ideal early biomarker for HIRI. As such, these experiments suggested that MB-Gly could function as a powerful platform for the real-time imaging and dynamic monitoring of HOCl during the whole process of HIRI.

We then evaluated the efficacy of several HIRI drugs. A series of HIRI drugs, including astragaloside IV (As-IV), tetramethylpyrazine (TMP), all-trans retinoic acid (ATRA), edaravone, NAC and melatonin, were chosen to assess the pharmacological function using MB-Gly. As-IV can improve liver parenchymal cell injury during HIRI by down-regulating the levels of tumor necrosis factor-α (TNF-α) and the expression of nuclear factor-κB (NF-κB) and up-regulating the expression of the glucocorticoid receptor (GR).^46^ TMP inhibits the formation of neutrophil extracellular traps (NETs) by inactivating NADPH oxidase, thereby alleviating HIRI.^47^ ATRA enhances the activity of superoxide dismutase (SOD), reducing the levels of oxidative stress and preventing HIRI.^28^ As a potent scavenger of hydroxyl radicals, edaravone reduces oxidative stress and inhibits the subsequent damaging inflammation by decreasing the expression of inflammatory cytokines and adhesion molecules, which ultimately alleviates HIRI.^48^ Melatonin can up-regulate the genes of antioxidant enzymes such as SOD, glutathione peroxidase (GSH-Px) and catalase (CAT), reduce the oxidative stress damage of hepatocytes, and decrease the production of proinflammatory cytokines and chemokines.^49^ However NAC reduces ROS release during HIRI by maintaining adequate glutathione concentrations.^50^ After pretreatment with the above drugs (500 μM) respectively, we established a HIRI model in hepatocytes and incubated with MB-Gly for evaluation. Significantly, 2.93-fold fluorescence enhancement was detected in HIRI cells compared with the control group. However, the red fluorescence of MB-Gly in HIRI hepatocytes after pretreatment with NAC and melatonin was significantly reduced. The red fluorescence was 1.58-fold and 1.24-fold for NAC and melatonin pretreated HIRI hepatocytes in comparison to the control group, respectively, indicating that the therapeutic effects of NAC and melatonin were superior to those of other four drugs (Fig. 2C and D). Furthermore, the dynamic fluorescence imaging of endogenous HOCl in hepatocytes upon treatment with several drugs in the processes of HIRI was performed to confirm their therapeutic effect (Fig. S5†). Taken together, MB-Gly facilitated the real-time dynamic imaging of the HIRI process, paving ways for screening potential HIRI drugs in hepatocytes.

### Therapeutic effects of MB-Gly on HIRI hepatocytes

A series of pathological events, including mitochondrial dysfunction, imbalance of energy metabolism, Ca^2+^ overload, oxidative stress, up-regulation of pro-inflammatory cytokines, and lipid peroxidation, are involved in HIRI.^13,17^ To comprehensively assess the therapeutic effect of MB-Gly during HIRI, we determined HIRI biomarkers after treatment with MB-Gly, including alanine aminotransferase (ALT), aspartate aminotransferase (AST), lactate dehydrogenase (LDH), TNF-α, adenosine triphosphate (ATP), malondialdehyde (MDA), ROS, and Ca^2+^ (Fig. 3N).^51,52^ Hepatocytes were divided into the normal group, HIRI group, glycine-treated HIRI group and MB-Gly-treated HIRI group. As shown in Fig. 3D, LDH release in HIRI cells was enhanced 4.2 times compared with that in the normal group, indicating that serious damage of the HIRI liver cell membrane occurred and resulted in LDH release. 0.40-fold and 0.30-fold decreases of LDH release were detected after treatment with glycine and MB-Gly in HIRI hepatocytes, respectively. Fig. 3E indicates that the TNF-α release of HIRI hepatocytes after MB-Gly treatment decreased from 42.7 pg mL^−1^ to 13.0 pg mL^−1^, indicating that MB-Gly could effectively reduce the pro-inflammatory cytokine and relieve hepatocyte injury. More importantly, the AST and ALT levels of the four groups of cells were evaluated. As depicted in Fig. 3F and G, the AST and ALT contents of HIRI hepatocytes after MB-Gly treatment were decreased to 28.2 IU per gprot and 15.8 IU per gprot, respectively. We found that the therapeutic effect of MB-Gly was superior to that of the same concentration of glycine, which may be because the cleavage product MB exhibits both anti-inflammatory and antioxidant effects.^39,53^ Collectively, these results illustrated that MB-Gly is a promising therapeutic tool that can down-regulate the release of LDH, TNF-α and intracellular ALT and AST.

Due to Ca^2+^ overload, imbalance of energy metabolism, oxidative stress and lipid peroxidation play crucial roles in the progression of HIRI, so we investigated the therapeutic effect of MB-Gly using key biomarkers of pathological events, including, Ca^2+^, ATP, ROS and MDA.^13^ We found that the levels of Ca^2+^ in HIRI hepatocytes were significantly increased, while fluctuations of Ca^2+^ levels in hepatocytes decreased after treatment with MB-Gly (Fig. 3H). We anticipated that the glycine cleavage product from MB-Gly could prevent the opening of Ca^2+^ channels in the cell membrane by binding to glycine receptors of MB-Gly-treated HIRI cells, downregulating Ca^2+^ levels and preventing Ca^2+^ overload.^54^ Moreover, as shown in Fig. 3I, intracellular ATP levels of HIRI hepatocytes were 22% lower than those of the normal group, implying energy depletion in the HIRI process. However, upon treatment with MB-Gly and glycine, recovery of ATP concentrations was detected, suggesting that MB-Gly could help maintain the energy supply. More importantly, enhanced MDA (Fig. 3J) and ROS (Fig. 3K and S6†) levels during HIRI were detected in HIRI, demonstrating that lipid peroxidation and oxidative stress synergistically occurred in HIRI. However MDA and ROS concentrations were effectively reduced in the MB-Gly and glycine-treated HIRI groups. Mitochondrial membrane potential decline is a hallmark of early apoptosis in HIRI. Flow cytometric analysis was conducted to assess the mitochondrial membrane potential of four groups of hepatocytes. Fig. S7† indicates that MB-Gly could reverse the decline of mitochondrial membrane potential during HIRI. Finally, to gain insight into the effect of MB-Gly on hepatocytes in HIRI, flow cytometry was again used to characterize apoptosis and necrosis. As shown in Fig. 3L, apoptotic cells accounted for 5.14% in the normal group and 45.4% in the HIRI group. Necrotic cells were 4.38% of the normal group and 38.04% of the HIRI group, suggesting obvious apoptosis and necrosis of HIRI cells. However, after treatment with glycine and MB-Gly during HIRI, the proportion of apoptotic and necrotic cells decreased significantly. Fig. 3M indicates that MB-Gly exhibits significant antioxidant, anti-inflammatory and anti-apoptotic effects on HIRI hepatocytes by comparing multiple biomarker levels, exhibiting better protective effects than glycine and thereby alleviating hepatocyte damage. As such, the above results confirm that MB-Gly enhanced HOCl clearance on cleavage of the urea bond due to the inherent antioxidant and anti-inflammatory activities of the products MB and glycine, thereby exhibiting excellent therapeutic function in HIRI hepatocytes.

### *In vivo* imaging diagnosis based on HOCl activation by MB-Gly

**Fig. 4:**
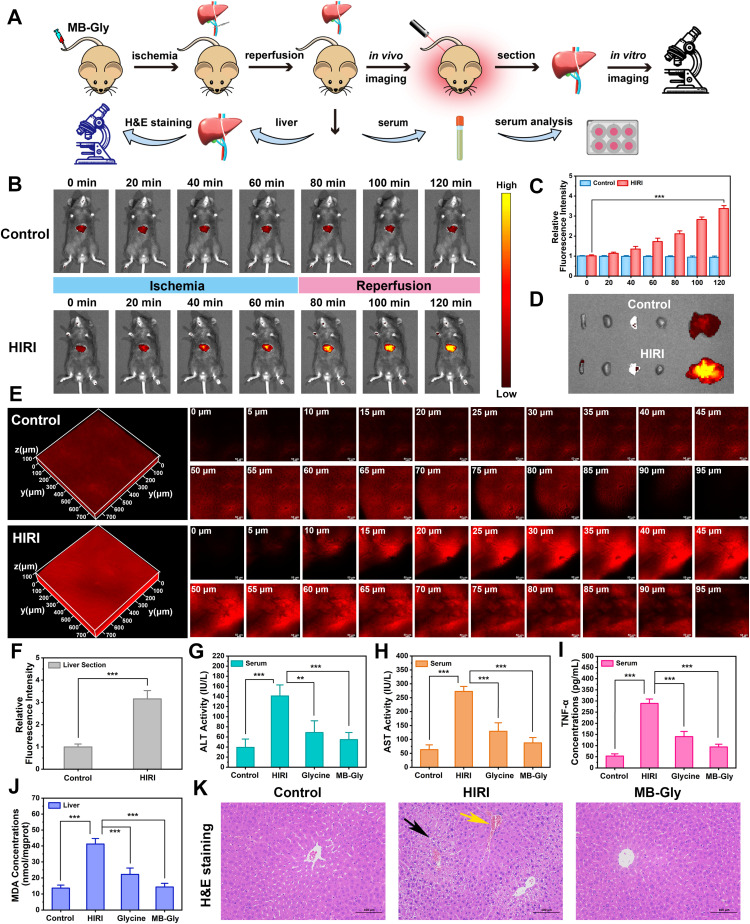
Fluorescence imaging and the therapeutic effect of MB-Gly on HIRI mice. (A) Schematic diagram of the procedure of using MB-Gly for the imaging and treatment of HIRI mice. (B) Dynamic fluorescence imaging of HOCl *in vivo* during the HIRI process. *λ*_ex_ = 620 nm, *λ*_em_ = 670 nm. (C) Relative fluorescence intensity of livers in the control group and HIRI group mice. (D) Fluorescence imaging of major organs *in vitro*. *λ*_ex_ = 620 nm, *λ*_em_ = 670 nm. (E) 3D fluorescence imaging of HOCl in liver sections of mice. *λ*_ex_ = 638 nm, *λ*_em_ = 643–775 nm. (F) Relative fluorescence intensity of liver sections. (G) Serum ALT content. (H) Serum AST content. (I) Serum TNF-α content. (J) MDA concentrations in liver tissues. (K) H&E staining of liver tissues.

Encouraged by the above results where MB-Gly played both diagnostic and therapeutic roles in HIRI hepatocytes, the feasibility of the fluorescence visualization and treatment of liver injury in living mice by MB-Gly was investigated (Fig. 4A). The control group and HIRI group mice were intravenously injected with MB-Gly to monitor HOCl-related red fluorescence signals in the livers, respectively. HIRI mouse models were established by simulating liver surgery in C57 mice.^26^ In the HIRI group, the portal vein and hepatic artery of the middle and left lateral lobes of the livers were clamped with microvascular clamps for 1 h, resulting in 70% liver ischemia. Subsequently, the vascular clamp was opened for 1 h of reperfusion. The livers of the control group were exposed as control. As shown in Fig. 4B and C, compared with normal mice, 3.4-fold red fluorescence enhancement was detected in the livers of the HIRI model mice, indicating that excessive HOCl could induce red fluorescence emission of MB-Gly in HIRI mice livers. The biological distribution of MB-Gly was then investigated. Major organs, including the heart, liver, spleen, lung, and kidney, of normal mice and HIRI mice were removed and *in vitro* fluorescence imaged. Fig. 4D indicates that MB-Gly was mainly accumulated in the livers of the control and HIRI group mice. The livers of the normal group and HIRI group mice were sectioned and the red fluorescence signal determined using confocal fluorescence microscopy. As shown in Fig. 4E, MB-Gly allowed for visualization of HOCl levels in the liver tissues of mice at a depth of 93 μm using 3D images. Moreover, the livers of HIRI mice exhibited 3.2 times more red fluorescence than that of normal mice, which facilitated the distinction between normal and HIRI mice (Fig. 4F).

### Therapeutic effect of MB-Gly on HIRI mice

Furthermore, the *in vivo* therapeutic effect of MB-Gly on C57 mice was investigated. The mice were randomly divided into the control group, HIRI model group, MB-Gly-treated HIRI group and glycine-treated HIRI group. The mice in these four groups were then independently intravenously injected with saline (control group), saline (HIRI group), MB-Gly (MB-Gly-treated HIRI group) and glycine (glycine-treated HIRI group). After the intravenous injection, HIRI models were established in the HIRI model group, MB-Gly-treated HIRI group and glycine-treated HIRI group. The livers of normal mice were exposed as a control. For these four groups of mice, the levels of ALT, AST and TNF-α in serum and the contents of MDA and ATP in the liver tissues were comprehensively evaluated. As shown in Fig. 4G and H, ALT and AST contents in the serum of the HIRI group mice were significantly increased, while the MB-Gly-treated HIRI mice exhibited reduced concentrations of ALT and AST. Similarly, after treatment with MB-Gly, TNF-α levels of the serum in mice were decreased from 289.0 pg mL^−1^ to 93.9 pg mL^−1^ (Fig. 4I), which illustrated the anti-inflammatory effect of MB-Gly. In addition, a decrease of the MDA content was detectable after MB-Gly treatment, implying the inhibition of lipid peroxidation (Fig. 4J). Notably, ATP levels recovered in the livers of HIRI mice upon administration of MB-Gly (Fig. S8†), suggesting the alleviation of energy consumption in HIRI processes by MB-Gly.

To further confirm the rehabilitation effect of MB-Gly, liver tissue slices of the control group, HIRI group, and MB-Gly-treated HIRI group were taken for histopathological analysis (Fig. 4K). Hematoxylin and eosin (H&E) staining showed that hepatic lobules in the normal group were clearly demarcated and arranged regularly. There was no obvious expansion or compression of hepatic sinuses. In addition, no obvious abnormalities in the portal area between adjacent hepatic lobules were observed. However in the liver tissues of HIRI mice, mild steatosis of hepatocytes around the central vein (black arrow) was clearly seen in the liver tissue. Small circular vacuoles were seen in the cytoplasm, portal venous congestion was seen in the portal area (yellow arrow), and the vascular lumen was full of red blood cells. Surprisingly, no significant injury and inflammation was observed in the MB-Gly-treated HIRI group. In addition, H&E staining of major organs, including the heart, spleen, lung, and kidney, in the three groups of mice was also conducted to interrogate the biotoxicity of MB-Gly *in vivo*. No significant injury was observed in the heart, spleen, lung, and kidney of these three groups (Fig. S9†), further validating the good biocompatibility of MB-Gly. Altogether, these results confirmed that MB-Gly exhibited versatile action toward HIRI *in vivo* and could offer not only high-fidelity imaging in HIRI but also effective treatment of HIRI.

### RNA sequencing and gene expression analysis

**Fig. 5:**
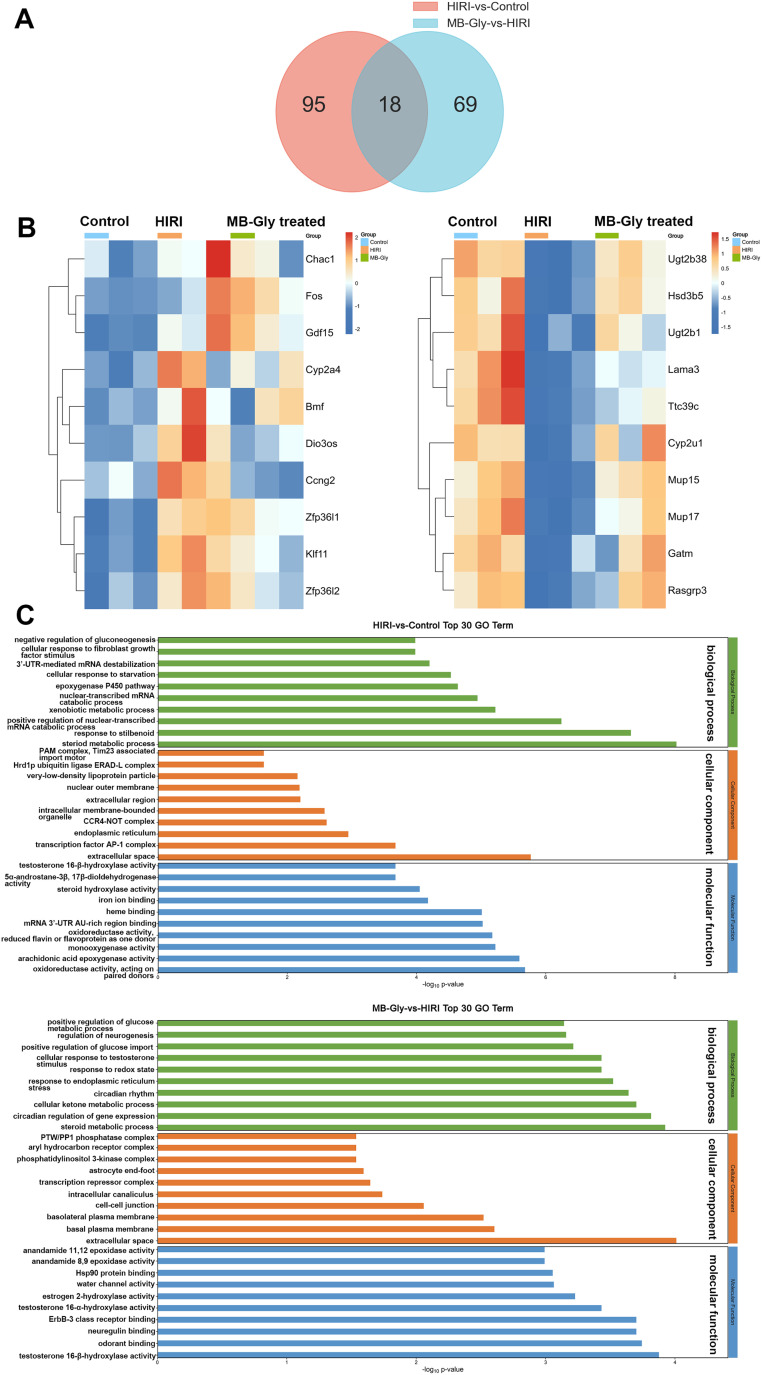
Differential analysis of RNA expression in mouse livers. (A) Venn diagram showing DEGs in the HIRI group *vs.* control group and the HIRI group *vs.* MB-Gly-treated HIRI group. (B) Cluster analysis of DEGs between the control group, HIRI group and MB-Gly-treated HIRI group. (C) GO terms of DEGs in the control group, HIRI group and MB-Gly-treated HIRI group. Green: biological process; orange: cellular component; blue: molecular function.

The above experiments confirmed the protective role of MB-Gly in HIRI mice, and we then investigated the mechanism of action of MB-Gly in mice livers during HIRI. For the liver tissues of the control group, HIRI group and MB-Gly-treated HIRI group, RNA sequencing and differential gene analysis were performed. The differentially expressed genes (DEGs) were screened according to thresholds of *q* value < 0.05 and |log_2_ FC| > 1. We found that a total of 113 DEGs were identified in the livers of the HIRI group compared with the control group, wherein 71 were up-regulated and 42 were down-regulated (Fig. 5A). In addition, a total of 87 DEGs were identified in the MB-Gly-treated HIRI group compared with the HIRI group, of which 46 were up-regulated and 41 were down-regulated (Fig. 5A and S13†). Subsequently, clustered heat map analysis was performed based on the DEGs to analyze the effect of MB-Gly on the HIRI mice at the gene level (Fig. 5B). The expression of pro-apoptotic-related genes (*e.g.*, Bmf and Chac1),^55^ the oxidative stress-related gene (*e.g.*, Cyp2a4),^56^ inflammation-related genes (*e.g.*, Fos and Gdf15),^57^ and cellular senescence-related genes (*e.g.*, Zfp36l1 and Zfp36l2) was up-regulated in mice undergoing the HIRI injury process, whereas the expression of the above-mentioned genes was decreased in MB-Gly-treated HIRI mice. Meanwhile, the expression of the anti-inflammatory factor-related gene (*e.g.*, Rasgrp3)^58^ and metabolism-related genes (*e.g.*, Cyp2u1 and Gatm) was decreased in HIRI mice,^59^ while the expression of Rasgrp3, Cyp2u1 and Gatm was increased after treatment with MB-Gly. These results suggest that MB-Gly influences the developmental process of HIRI by inhibiting pro-apoptotic and oxidative stress-related gene expression while activating anti-inflammatory and metabolism-related gene expression.

To further analyze the potential functions of DEGs between the control group, HIRI group, and MB-Gly-treated HIRI group, gene ontology (GO) analysis was performed. GO terms of DEGs were selected for analysis in each of the three sections: biological process (BP), cellular component (CC) and molecular function (MF) (Fig. 5C). The experimental results indicated that the DEGs between the control group and HIRI group were mainly enriched in the steroid metabolic process during BP. DEGs between the MB-Gly-treated HIRI group and HIRI group were enriched in the steroid metabolic process in terms of BP. From the Kyoto Encyclopedia of Genes and Genomes (KEGG) analysis, the down-regulated DEGs in the HIRI group were mainly involved in retinol metabolism, steroid hormone biosynthesis and the cytochrome P450 pathway, and their enrichment was compared with that of the control group (Fig. S14†). In addition, the upregulated DEGs were distributed in the p53, TNF and FoxO signaling pathways associated with inflammation and apoptosis.^60,61^ However DEGs enriched in inflammatory-related and apoptosis-related pathways, such as MAPK and p53 signaling pathway, exhibited decreasing trends in the MB-Gly-treated HIRI group (Fig. S15†).^62^ Compared with the HIRI group, the up-regulated DEGs in the MB-Gly-treated HIRI group were mainly enriched in the metabolism-related processes such as the cytochrome P450 pathway, butanoate metabolism and steroid hormone biosynthesis. The above results indicated that the relief of HIRI provided by MB-Gly is mainly due to increased expression of genes related to metabolism and decreased expression of genes related to inflammation pathology.

## Conclusions

In summary, a smart multifunctional system MB-Gly for the early diagnosis of HIRI at the molecular level and targeted treatment was developed. Excessive HOCl during HIRI induced rapid cleavage of the urea bond in MB-Gly, leading to the synergistic production of MB and glycine, which facilitated fluorescence monitoring and precision therapy of HIRI. MB-Gly was shown to be a versatile tool for HOCl evaluation in HIRI, exhibiting high sensitivity, superb selectivity, pH insensitivity and good photostability. Using the excellent spatiotemporal resolution of MB-Gly, MB-Gly could successfully visualize HOCl levels in hepatocytes and live mouse livers during HIRI and evaluate the efficacy of potential HIRI drugs. Through the joint evaluation of HIRI related biomarkers, MB-Gly exhibited powerful therapeutic effects on hepatocytes and mice suffering from HIRI. As such, our multifunctional platform was able to track and monitor the evolution of liver function using NIR fluorescence. RNA sequencing confirmed the therapeutic effect exerted by MB-Gly by affecting the expression of genes associated with apoptosis, oxidative stress, inflammation and metabolism. This study provided a “three-in one” strategy for developing integrated diagnostic and treatment tools, paving the way for the early diagnosis and targeted therapy of oxidative stress-related diseases.

## Data availability

The data that support the findings of this study are available in the ESI† of this article.

## Author contributions

J. H. L. and D. N. Y. contributed equally. The manuscript was written through contributions of all authors. All authors have given approval to the final version of the manuscript.

## Conflicts of interest

There are no conflicts to declare.

## Supplementary Material

SC-OLF-D4SC04962D-s001
