# Supplementary material for: A multifunctional “three-in-one” fluorescent theranostic system for hepatic ischemia–reperfusion injury
Source: Chem Sci. 2024 Nov 13;15(47):19820–33. doi: 10.1039/d4sc04962d (PMC11575585; doi:10.1039/d4sc04962d)
Supplement: SC-015-D4SC04962D-s001 [file SC-015-D4SC04962D-s001.pdf]

Supplementary Information (SI) for Chemical Science.
This journal is © The Royal Society of Chemistry 2024

A Multifunctional "Three-in-One" Fluorescent Theranostic System for Hepatic Ischemia−Reperfusion Injury

Jihong Liu,[ae#] Dongni Yin,[a#] Wen Zhang,[a*] Xin Wang,[a] Tony D. James,[adf*] Ping Li,[ab*] and Bo Tang[ac*]

a. College of Chemistry, Chemical Engineering and Materials Science, Key Laboratory of Molecular and Nano Probes, Ministry of Education, Collaborative Innovation Center of Functionalized Probes for Chemical Imaging in Universities of Shandong, Institutes of Biomedical Sciences, Shandong Normal University, Jinan 250014, People's Republic of China.

b. College of Chemistry and Chemical Engineering, Northwest Normal University, Lanzhou 730070, People's Republic China.

c. Laoshan Laboratory, Qingdao 266237, People's Republic of China.

d. Department of Chemistry, University of Bath, Bath, BA2 7AY, UK.

e. Key Laboratory for Advanced Materials and Joint International Research Laboratory of Precision Chemistry and Molecular Engineering, Feringa Nobel Prize Scientist Joint Research Center, Shanghai Key Laboratory of Functional Materials Chemistry, Institute of Fine Chemicals, Frontiers Science Center for Materiobiology and Dynamic Chemistry, School of Chemistry and Molecular Engineering, East China University of Science and Technology, Shanghai 200237, People's Republic of China

f. School of Chemistry and Chemical Engineering, Henan Normal University, Xinxiang. 453007, People's Republic of China.

Email: zhangwen@sdnu.edu.cn, t.d.james@bath.ac.uk, lip@sdnu.edu.cn, tangb@sdnu.edu.cn

Table of Contents

Experimental Procedures S4
Materials and instruments S4
Preparation of ROS/RNS S4
Fluorescence spectra of MB-Gly toward HOCl S5
Fluorescence spectra of MB-Gly toward HOCl in the presence of other interferences ..S5
LOD calculation S5
HPLC Analysis S5
Cells culture S6
Cytotoxicity assays S6
Hepatic ischemia−reperfusion injury models in cells S6
Fluorescence imaging of HOCl in cells under external stimulus S6
Fluorescence imaging of HOCl in hepatocytes during HIRI S6
Fluorescence imaging for efficacy evaluation of HIRI drugs S7
Evaluation of therapeutic effect of MB-Gly on hepatocytes during HIRI S7
Hepatic ischemia−reperfusion injury models in mice S7
In vivo and *in vitro* fluorescence imaging of HOCl in mice during HIRI S8
3D fluorescence imaging of HOCl in liver sections of mice during HIRI S8
Evaluation of therapeutic effect of MB-Gly in mice during HIRI S8
H&E staining of major organs in mice S8
BCA protein assays S8
Aspartate aminotransferase activity assays S9
Alanine aminotransferase activity assays S9
Lactate dehydrogenase release determination S9
Tumor necrosis factor-α determination S10
ATP content assays S10
MDA content assays S11
ROS content assays S11
Ca^{2+} content assays S11
HPLC analysis with precolumn derivatization S11
RNA sequencing and data analysis S12
Annexin V-FITC cell apoptosis analysis S12
Measurement of mitochondrial membrane potential S12
Statistical analysis S13
Data availability S13
Results and Discussion S13
Synthesis of MB-Gly S13
Scheme S1. ....S13
Figure S1. ....S15
Figure S2. ....S16
Figure S3. ....S17
Figure S4. ....S18
Figure S5. ....S19
Figure S6. ....S20

Figure S7. ....S21
Figure S8. ....S22
Figure S9. ....S23
Figure S10. ....S24
Figure S11. ....S25
Figure S12. ....S26
Figure S13. ....S27
Figure S14. ....S28
Figure S15. ....S29
References...S30

Experimental Procedures

Materials and instruments

Methylene blue and glycine were purchased from Shanghai Macklin Biochemical Co., Ltd. Phorbol 12-myristate 13-acetate (PMA) was obtained from Shanghai Aladdin Biochemical Technology Co., Ltd. Lipopolysaccharide (LPS) was prepared from Beijing Solarbio Science & Technology Co., Ltd. 4-aminobenzoic acid hydrazide (ABH) was purchased from Adamas-beta (Shanghai). *N*-acetyl-L-cysteine (NAC) was purchased from Shanghai Aladdin Biochemical Technology Co., Ltd. Edaravone was obtained from Shanghai Aladdin Biochemical Technology Co., Ltd. Tetramethylpyrazine (TMP), all-trans retinoic acid (ATRA), astragaloside IV (As-IV), melatonin were obtained from Shanghai Macklin Biochemical Co., Ltd. Lactate Dehydrogenase (LDH) Release Assay Kit, Lipid Peroxidation MDA Assay Kit, Reactive Oxygen Species Assay Kit and Annexin V-FITC Cell Apoptosis Detection Kit were obtained from Beyotime. Tumor Necrosis Factor-α (TNF-α) ELISA kit was purchased from 4A Biotech Co., Ltd. Aspartate Aminotransferase (AST) Activity Assay Kit and Alanine Aminotransferase (ALT) Activity Assay Kit were purchased from Elabscience. ATP Contents Assay Kit was obtained from Nanjing Jiancheng Bioengineering Institute. Bicinchoninic acid (BCA) Protein Assay Kit (GenStar) was used. Mitochondrial Membrane Potential Assay Kit with JC-1 and Calcium ion fluorescent probe (Fura-2 Am) were purchased from Beyotime.

Absorption spectra were recorded on a UV-Visible spectrophotometer (Evolution 220, Thermo Scientific). Fluorescence spectra were obtained with a Hitachi F-4700 fluorescence spectrophotometer. CCK-8 assay was performed using a Triturus microplate reader. Confocal imaging was performed on Leica SP8 high-resolution fluorescence microscope. Fluorescence imaging in mice was performed on a Caliper IVIS Lumina Series III. Mice liver slices of 100 μm thickness were obtained with cryostat (Leica CM1950). Hematoxylin-eosin (H&E) staining images were obtained using an optical microscope (Nikon, Eclipse Ci-L). The mass spectra were obtained using the Bruker Maxis ultra-high-resolution-TOF MS system. ^{1}H NMR spectra were obtained at 400 MHz using Bruker NMR spectrometers, and ^{13}C NMR spectra were recorded at 100 MHz. HPLC analysis was carried out on a Shimadzu LC-16 system equipped with SPD-16 UV−vis detector. The transcriptome sequencing results were acquired by llumina Novaseq 6000 platform (OE Biotech Co., Ltd. Shanghai). Liquid chromatography-mass spectrometry (LC-MS, Thermofisher U3000-Q Exactive Focus, USA) was used to analyze the release of glycine.

Preparation of ROS/RNS

$O_2^{\bullet-}$

$O_2^{\bullet-}$ was produced from KO_2 in dry DMSO by an ultrasonic method. The concentration of $O_2^{\bullet-}$ was determined from the absorption at 250 nm ($\varepsilon = 2682\ M^{-1}\ cm^{-1}$).

$ONOO^-$

0.6 M $NaNO_2$, 0.6 M HC1 and 0.7 M H_2O_2 were added simultaneously to a 3 M NaOH solution at 0 ºC. The

concentration of $ONOO^-$ was determined using extinction coefficient of 1670 M^{-1} cm^{-1} at 302 nm in 0.1 M NaOH (aq.).

H_2O_2

H_2O_2 solutions were accessed by dilution of 30% hydrogen peroxide aqueous solution, the concentration was determined from the absorption at 240 nm (ε = 43.6 M^{-1} cm^{-1}).

TBHP

TBHP solutions were accessed by dilution of 70 % *tert*-butyl hydroperoxide aqueous solution.

$^{\bullet}OH$

$^{\bullet}OH$ (hydroxyl radical) was generated by the Fenton reaction of $FeCl_2$ with H_2O_2 (1:6) in deionized water.[1]

NO

NO (Nitric oxide) was obtained from a stock solution prepared by sodium nitroprusside (SNP).

$^1O_2$

$^1O_2$ (Singlet oxygen) was prepared by the reaction of 10 mM of NaClO and 10 mM of H_2O_2 in PBS buffer solutions (pH = 7.4).

Fluorescence spectra of MB-Gly toward HOCl

Various concentrations of HOCl (0–38 μM) were added to PBS buffer solution (10 mM, pH = 7.40) containing MB-Gly (25 μM), and the fluorescence spectrum was recorded after incubation for 20 min. λ_{ex} = 620 nm.

Fluorescence spectra of MB-Gly toward HOCl in the presence of other interferences

38 μM HOCl was added to PBS buffer solution (10 mM, pH = 7.40) containing MB-Gly (25 μM) and other interfering substances (10 mM H_2O_2, 100 μM $^1O_2$, 100 μM TBHP, 100 μM $^{\bullet}OH$, 100 μM $O_2^{\bullet-}$, 50 μM $NO^{\bullet}$, 50 μM $ONOO^-$, Blank, 10 mM K^+, 10 mM Na^+, 200 μM Ca^{2+}, 200 μM Mg^{2+}, 200 μM Zn^{2+}, 200 μM Fe^{2+}, 200 μM Fe^{3+}, 200 μM Cu^{2+}, 1 mM Pro, 1 mM Ser, 1mM His, 1 mM Lys, 1 mM Arg, 1 mM Asn, 1 mM Gln, 1mM Gly, 1 mM Asp, 1 mM Met, 1 mM Cys, 1 mM Hcy). The fluorescence spectrum was recorded after incubation for 20 min. $\lambda_{ex/em}$ = 620/687 nm.

LOD calculation

The limit of detection (LOD) was calculated using the well-established method (LOD = 3σ/K), where K is slope of the calibration curve, σ represents is the standard deviation of the blank sample (11 times) of F_{687} for MB-Gly without addition of HOCl.[2] (Note F_{687} refer to the Fluorescence (F) emission wavelength peaks at 687 nm).

HPLC Analysis

For HPLC analysis, deionized water was used as eluent A and methanol as eluent B. HPLC conditions: 0-1 min: 50% of A, 50% of B; 1-5 min: 69% of A, 31% of B; 5-13 min: 50% of A, 50% of B; 13-35 min: 12.5% of A, 87.5% of B. The injection volume was 10 μL. The parameters of the HPLC-MS analytical column used were C18-WR, 5 μm, 4.6 mm × 150 mm (GL Sciences). The flow rate is 0.8 mL/min.

Cells culture

Human hepatocytes (HL-7702) were purchased from the Cell Bank of the Chinese Academy of Sciences (Shanghai, China). Hepatocytes were cultured in high-glucose DMEM supplemented with 10 % fetal bovine serum, 1 % penicillin and 1 % streptomycin (w v^{-1}) at 37 °C in a 5 % CO_2/95 % air MCO-15AC incubator (SANYO, Tokyo, Japan).

Cytotoxicity assays

Cell Counting Kit-8 (CCK-8) assays were carried out to evaluate the toxicity of MB-Gly, glycine and methylene blue. Hepatocytes (10^6 cells mL^{-1}) were seeded into 96-well microtiter plates with total volumes of 200 μL $well^{-1}$. After 24 h of incubation, various concentrations of MB-Gly, glycine and methylene blue (0 M, 1×10^{-9} M, 1×10^{-8} M, 1×10^{-7} M, 1×10^{-6} M, 1×10^{-5} M, 1×10^{-4} M and 1×10^{-3} M) were added, and the hepatocytes were cultured for another 24 h. Afterwards, 10 μL of CCK-8 solution was added to each well. After 4 h of incubation, the absorbance at 450 nm was measured using a Triturus microplate reader.

Hepatic ischemia−reperfusion injury models in cells

Hepatic ischemia−reperfusion injury (HIRI) cell models were established by oxygen-glucose-serum deprivation/reperfusion. For the phase of ischemia, HL-7702 cells were cultured in DMEM (without glucose and serum) and deoxygenated sodium dithionite (0.5 mM) for 20 min or 40 min of ischemia. For the phase of subsequent reperfusion, these cells were incubated with high glucose and serum DMEM (standard DMEM) in a 5 % CO_2 and 95 % O_2 atmosphere for 20 min, 40 min or 60 min of reperfusion after 40 min ischemia.

Fluorescence imaging of HOCl in cells under external stimulus

HL-7702 cells were divided into five groups. The control group cells were stained with MB-Gly (50 μM) and Hoechst 33342 (1 μg/mL) for 20 min. The LPS stimulated group cells were incubated with LPS (1.0 μg/mL) for 30 min, then stained with MB-Gly (50 μM) and Hoechst 33342 (1 μg/mL) for 20 min. The LPS & PMA stimulated group cells were incubated with LPS (1.0 μg/mL) and PMA (1.0 μg/mL) for 30 min, then stained with MB-Gly (50 μM) and Hoechst 33342 (1 μg/mL) for 20 min. The ABH stimulated group cells were pretreated with ABH (500 μM) for 30 min, followed by adding LPS (1.0 μg/mL) and PMA (1.0 μg/mL) for 30 min and then stained with MB-Gly (50 μM) and Hoechst 33342 (1 μg/mL) for 20 min. The NAC stimulated group cells were pretreated with NAC (500 μM) for 30 min, followed by adding LPS (1.0 μg/mL) and PMA (1.0 μg/mL) for 30 min and then stained with MB-Gly (50 μM) and Hoechst 33342 (1 μg/mL) for 20 min. The cell culture medium of each group was removed, and all cells were washed with 1.0 mL of PBS three times before fluorescence imaging. One-photon confocal images were taken using a Leica SP8 high-resolution fluorescence microscope equipped with the Leica Application Suite X software package.

Fluorescence imaging of HOCl in hepatocytes during HIRI

In order to perform fluorescence imaging of HOCl during the process of HIRI, HL-7702 cells were divided into six groups. Control group hepatocytes were cultured with high glucose and serum DMEM in a 5 % CO_2

and 95 % O_2 atmosphere. The 20 min of ischemia group hepatocytes were cultured in DMEM (without glucose and serum) and deoxygenated sodium dithionite (0.5 mM) for 20 min. The 40 min of ischemia group hepatocytes were cultured in DMEM (without glucose and serum) and deoxygenated sodium dithionite (0.5 mM) for 40 min. For 40 min of ischemia followed by 20 min of reperfusion group, hepatocytes were cultured in DMEM (without glucose and serum) and deoxygenated sodium dithionite (0.5 mM) for 40 min. Afterwards, cells were incubated with high glucose and serum DMEM in a 5 % CO_2 and 95 % O_2 atmosphere for 20 min. For 40 min of ischemia followed by 40 min of reperfusion group, hepatocytes were cultured in DMEM (without glucose and serum) and deoxygenated sodium dithionite (0.5 mM) for 40 min. Afterwards, cells were incubated with high glucose and serum DMEM in a 5 % CO_2 and 95 % O_2 atmosphere for 40 min. For 40 min of ischemia followed by 60 min of reperfusion group, hepatocytes were cultured in DMEM (without glucose and serum) and deoxygenated sodium dithionite (0.5 mM) for 40 min. Afterwards, cells were incubated with high glucose and serum DMEM in a 5 % CO_2 and 95 % O_2 atmosphere for 60 min. All the groups were treated with 50 μM MB-Gly and Hoechst 33342 (1 μg/mL) for 20 min before confocal imaging.

Fluorescence imaging for efficacy evaluation of HIRI drugs

To investigate the efficacy of HIRI drugs, HL-7702 cells were pretreated with astragaloside IV (500 μM), tetramethylpyrazine (500 μM), ATRA (500 μM), edaravone (500 μM), NAC (500 μM), melatonin (500 μM) for 1 h, respectively. Subsequently, the cells were cultured with DMEM (without glucose and serum) and deoxygenated sodium dithionite (0.5 mM) for 40 min, followed by incubation with standard DMEM in a 5 % CO_2 and 95 % O_2 atmosphere for 60 min. All the groups were treated with 50 μM MB-Gly for 20 min before confocal imaging.

Evaluation of therapeutic effect of MB-Gly on hepatocytes during HIRI

HL-7702 cells were pretreated with MB-Gly (50 μM) or glycine (50 μM) for 1 h, respectively. Then, the MB-Gly-pretreated HIRI cells and glycine-pretreated HIRI cells were cultured with DMEM (without glucose and serum) and deoxygenated sodium dithionite (0.5 mM) for 40 min, followed by incubation with standard DMEM in a 5 % CO_2 and 95 % O_2 atmosphere for 60 min. Control group hepatocytes were cultured with high glucose and serum DMEM in a 5 % CO_2 and 95 % O_2 atmosphere. HIRI group were cultured in DMEM (without glucose and serum) and deoxygenated sodium dithionite (0.5 mM) for 40 min. Afterwards, cells were incubated with high glucose and serum DMEM in a 5 % CO_2 and 95 % O_2 atmosphere for 60 min. Finally, cell culture medium and cells of control group, HIRI group, MB-Gly-pretreated HIRI group and glycine-pretreated HIRI group were collected for further biomarker analysis.

Hepatic ischemia−reperfusion injury models in mice

Six-week-old C57 mice (males) were used. A HIRI mouse model was established by simulating liver surgery. The mice in HIRI group were given a laparotomy to expose the liver, and hepatic ischemia was induced by clamping the portal vein and hepatic artery of the median and the left lateral lobes of the liver with a microvessel clip for 1 h, which induced partial (70 %) liver ischemia. Subsequently, the vascular clamp was opened for 1 h of reperfusion after 1 h of ischemia. For normal group, the liver was exposed as a control.

During the laparotomy surgery, the mice were anesthetized by inhaling isoflurane. All animal experiment methods were approved by Animal experiment ethical review committee of Shandong Normal University (Application number: AEECSDNU2024065).

***In vivo* and *in vitro* fluorescence imaging of HOCl in mice during HIRI**

MB-Gly with a dose of 100 μM was intravenously injected into control group mice and HIRI group mice. After 1 h, the mice in the control group were subjected to a laparotomy and the livers were exposed. For HIRI group, the portal vein and hepatic artery of the median and the left lateral lobes of the liver were clamped with a microvessel clip for 1 h. Subsequently, the vascular clamp was opened for 1 h of reperfusion. Subsequently, all group mice were transferred for *in vivo* imaging. After *in vivo* imaging, the mice were sacrificed and the major organs were removed for *in vitro* fluorescence imaging, including the hearts, livers, spleens, lungs and kidneys.

3D fluorescence imaging of HOCl in liver sections of mice during HIRI

MB-Gly with a dose of 100 μM was intravenously injected into control group mice and HIRI group mice. After 1 h, the mice in the control group were subjected to a laparotomy and the livers were exposed. For HIRI group, the portal vein and hepatic artery of the median and the left lateral lobes of the liver were clamped with a microvessel clip for 1 h. Subsequently, the vascular clamp was opened for 1 h of reperfusion. Subsequently, the livers of all groups were removed and sectioned using cryostat (Leica CM1950). Finally, the liver sections were imaged using a Leica SP8 high-resolution fluorescence microscope.

Evaluation of therapeutic effect of MB-Gly in mice during HIRI

To evaluate the therapeutic effect of MB-Gly in mice during HIRI, the mice were divided to four groups. MB-Gly-treated HIRI group and glycine-treated HIRI group mice were intravenously injected with MB-Gly (100 μM) and glycine (100 μM), respectively. After 1 h of injection, MB-Gly-treated HIRI group and glycine-treated HIRI group mice then underwent the HIRI process. The livers of the control group were exposed. The serum and liver tissues of the control group, HIRI group, MB-Gly-treated HIRI group and glycine-treated HIRI group were collected for further biomarkers analysis.

H&E staining of major organs in mice

The C57 mice were divided into three groups consisting of the control group, HIRI group and MB-Gly-treated HIRI group. MB-Gly-treated HIRI group was intravenously injected with MB-Gly (100 μM) and underwent the HIRI process. The liver tissues of control group, HIRI group and MB-Gly-treated HIRI group were collected to identify the histological changes. The liver tissues were fixed with 4% paraformaldehyde, and dehydrated, embedded, sectioned and stained by hematoxylin and eosin. Finally, the sections were imaged using an optical microscope (Nikon, Eclipse Ci-L).

BCA protein assays

The BCA protein assays were carried out by BCA Protein Assay Kit (GenStar). The principle of BCA

determination method is that Cu^{2+} can be reduced to Cu^{+} by protein under alkaline conditions, and Cu^{+} binds with the BCA reagent to form a purple complex. By measuring the absorbance of samples at 562 nm and comparing with the standard curve of reference samples, the protein concentration of the sample to be measured can be calculated. The hepatocytes and liver tissues samples under various treatment were collected and placed in ice-water bath for homogenate crushing. 20 μL of reference sample and hepatocytes samples were added in microplate, respectively. 200 μL of BCA working solution was added in each well and vibrated to mix sufficiently. The microplate was covered and incubated for 30 min at 37 °C. After cooling to room temperature, the absorbance at 562 nm was measured using a Triturus microplate reader. The protein concentrations of samples were determined according to the standard curve of reference samples and the dilution ratio of samples.

Aspartate aminotransferase activity assays

Aspartate aminotransferase (AST) activity assays were performed using an AST Activity Assay Kit (Elabscience). AST catalyzes the reaction between α-ketone glutaric acid and aspartic acid, generating glutamic acid and oxaloacetic acid. Oxaloacetic acid decarboxylates to pyruvic acid, and pyruvic acid and 2,4-dinitrobenzene hydrazine generates 2,4-dinitrobenzene hydrazone, which appears as reddish brown in alkaline solution. The AST activity per unit protein weight (IU/g protein) were calculated using the BCA Protein Assay kit and AST Activity Assay Kit. The hepatocytes and serum samples under various treatment were collected. 20 μL of substrate solution was added, followed by the addition of 5 μL of samples. The mixture of substrate solution and samples were mixed and placed at 37 °C for 30 min. Afterwards, 20 μL of chromogenic agent was added to the mixture and incubated at 37 °C for 20 min. 200 μL of Alkali reagent was added and mixed at room temperature for 15 min. The absorbance at 510 nm was measured. The levels of AST in samples were calculated according to the standard curve.

Alanine aminotransferase activity assays

Alanine aminotransferase activity assays (ALT) activity assays were performed using an ALT Activity Assay Kit (Elabscience). ALT catalyzes the reaction between alanine and α-ketone glutaric acid, producing glutamic acid and pyruvic acid. 2,4-dinitrobenzene hydrazine was added to stop this reaction, generating pyruvate phenylhydrazone which appears as reddish brown in alkaline conditions. The ALT activity per unit protein weight (IU/g protein) were calculated using the BCA Protein Assay kit and ALT Activity Assay Kit. The hepatocytes and serum samples under various treatment were collected. 20 μL of substrate solution was added, followed by the addition of 5 μL of samples. The mixture of substrate solution and samples were mixed and placed at 37 °C for 30 min. Afterwards, 20 μL of chromogenic agent was added in the mixture and incubated at 37 °C for 20 min. 200 μL of Alkali reagent was added and mixed at room temperature for 15 min. The absorbance at 510 nm was measured. The levels of ALT in samples were calculated according to the standard curve.

Lactate dehydrogenase release determination

Lactate dehydrogenase (LDH) release determination were performed using a LDH release assay kit

(Beyotime). After HIRI models were established in hepatocytes, the supernatants were collected and assayed for LDH. Under the action of LDH, nicotinamide adenine dinucleotide (NAD^+) is reduced to NADH, which react with 2-p-iodophenyl-3-nitrophenyl tetrazolium chloride to produce formazan under the catalytic reaction of diaphorase. The absorbance at 490 nm exhibits a linear increase with enhanced activity of LDH. Hepatocytes were seeded into a 96-well plate and divided into the control group and HIRI group. After treatment, the cell culture medium was collected, and LDH activity was measured using an LDH assay kit. An LDH releasing agent was provided in the kit and served as the positive control of total LDH release. Absorbance was measured at 490 nm using a microplate reader.

Tumor necrosis factor-α determination

Tumor necrosis factor-α (TNF-α) determination were performed using a TNF-α ELISA kit (4A Biotech Co., Ltd). The supernatants of hepatocytes and serum were collected and assayed for TNF-α. A monoclonal antibody specific for TNF-α was coated onto 96-well microtiter plates provided. TNF-α present in the samples or standard and the monoclonal anti TNF-α antibody conjugated to biotin were simultaneously incubated in microtiter plates. Following incubation unbound TNF-α was removed during a wash step. Streptavidin-HRP was added and bound to the biotinylated anti TNF-α. After incubation and wash step a substrate solution reactive with HRP was added to the wells. The reaction was terminated by adding stop solution. A yellow product was formed in proportion to the amount of TNF-α present in the sample. The absorbance was measured at 450 nm. The levels of TNF-α in samples were calculated according to the standard curve.

ATP content assays

The ATP content in hepatocytes and liver tissues per unit protein weight were calculated using the BCA Protein Assay kit and ATP Contents Assay Kit (Nanjing Jiancheng Bioengineering Institute). The principle of ATP determination method is that creatine kinase catalyzes ATP and creatine to produce creatine phosphate. For ATP measurement, the phosphomolybdic acid colorimetric method was used to detect the generation of creatine phosphate. The hepatocytes samples and liver tissues under various treatments were collected and placed in ice-water bath for homogenate crushing. The cell suspensions and tissues homogenate were heated in a boiling water bath for 10 min, vortex mixed for 1 min and centrifuged at 3,500 rpm for 10 min. To measure ATP concentrations, 30 µL supernatant, 100 µL substrate I, 200 µL substrate II and 30 µL accelerator were combined and incubated at 37 °C for 30 min. Then, 50 µL precipitant was added, and the mixture was centrifuged at 4,000 rpm for 5 min. A volume of 500 µL chromogenic fluids were combined with 300 µL supernatant and incubated at room temperature for 2 min. Finally, 500 µL termination fluid was added and incubated at room temperature for 5 min. A 200 µL volume of the mixture was added to a 96-well plate, and the absorbance values were measured at 636 nm on a Triturus microplate reader. The ATP concentrations were calculated as follows:

$$C_{ATP} = (A_{Sample} - A_{Control}) / (A_{Reference} - A_{Blank}) \times C_{Reference} \times N / Cpr$$

in which C_{ATP} is the concentration of ATP (nmol/mg protein), A_{Sample} is the absorbance of the sample, $A_{Control}$ was the absorbance of the control, $A_{Reference}$ is the absorbance of the reference, A_{Blank} is the absorbance of the

blank, $C_{Reference}$ is the concentration of the reference (1000 μmol/L), N is the sample dilution ratio before the determination, Cpr is the concentration of homogenate protein.

MDA content assays

The MDA contents in hepatocytes and liver tissues per unit protein weight were calculated using the BCA protein content assay kit and MDA Assay Kit. The above cell samples and liver tissue samples were placed in an ice-water bath for homogenate crushing. After lysis, the samples were centrifuged and the liquid supernatant was collected. 0.37 % of thiobarbituric acid (TBA) stock solution was prepared. MDA detection working solution was made through addition of TBA stock solution and TBA diluent and antioxidant. Reference samples were diluted and the concentration of 1 μM, 2 μM, 5 μM, 8 μM and 10 μM were prepared. Reference samples and samples to be tested are added with 200 μL MDA detection working solution and mixed. After mixing, the solution was heated at 100 °C for 15 min and cooled to room temperature. The solution was centrifuged for 10 min at 1000×g. 200 μL of the solution was added in 96-well plates and the absorptance of 532 nm was detected.

ROS content assays

The ROS content in hepatocytes were detected using Reactive Oxygen Species Assay Kit (S0033S, Beyotime). The cells samples were resuspended in DCFH-DA at 37 °C for 20 min and mixed at intervals of 3-5 min. The emission at 528 nm under 485 nm excitation was detected using a Triturus microplate reader.

Ca^{2+} content assays

The hepatocyte samples under various treatments were collected and incubated with Fura-2 AM (5 μM) at 37 ˚C for 45 min. After that, the suspension was washed three times using ice-cold PBS, then re-suspended in Krebs-Ringer buffer. Fluorescence value (F) was measured using fluorescence spectrophotometer under the excitation wavelength of 340 nm and the emission wavelength of 510 nm. The $[Ca^{2+}]_i$ were calculated using the following equation:

$$[Ca^{2+}]_i = K_d \times (F - F_{min}) / (F_{max} - F)$$

in which $[Ca^{2+}]_i$ is the concentration of intracellular Ca^{2+}, F is the fluorescence of the cells suspended in the Krebs-Ringer buffer, F_{max} is the fluorescence of the cells treated with 0.1% of Triton X-100, F_{min} is the fluorescence of the cells followed by addition of 10 mM EGTA (ethylene glycol tetra-acetic acid, pH 9.0), K_d is the dissociation constant of Fura-2 AM (224 nM at 37 °C).

HPLC analysis with precolumn derivatization

Glycine release from MB-Gly was determined by HPLC combined the with precolumn derivatization reaction. The derivatization procedure was carried out in the autosampler. The mobile phase consisted of two solvents. Solvent A was a mixture of 30 mM acetate buffer, tetrahydrofuran, and acetonitrile. Solvent B was a mixture of 147 mM acetate buffer, tetrahydrofuran, and acetonitrile. HPLC conditions: 0-27min, 92% of A, 8% of B; 27-30min, 50% of A, 50% of B. Samples were detected by a Thermo Fisher U3000-Q-Exactive high resolution liquid mass spectrometer. The column temperature was set at 40 °C. Detection was performed under the

excitation of 340 nm and the emission of 450 nm. The flow rate is 1 mL/min.

RNA sequencing and data analysis

Total RNA was extracted from liver tissues in control group, HIRI group, MB-Gly-treated HIRI group mice, respectively. The transcriptome sequencing and analysis were conducted by OE Biotech Co., Ltd. (Shanghai). Total RNA was extracted using the TRIzol reagent (Invitrogen, CA, USA). RNA purity and quantification were evaluated using the NanoDrop 2000 spectrophotometer (Thermo Scientific). RNA integrity was assessed using the Agilent 2100 Bioanalyzer (Agilent Technologies, Santa Clara, CA, USA). Then the libraries were constructed using VAHTS Universal V6 RNA-seq Library Prep Kit. IluminaS8 NovaSeq 6000 platform was used for sequencing after qualifying database inspection. Differential expression genes (DEGs) analysis was performed using the DESeq2 5. Q value < 0.05 and $|log_2FC| > 1$ was set as the threshold for significant DEGs.

Annexin V-FITC cell apoptosis analysis

Hepatocytes were seeded on six-well plates, cultured with high-glucose DMEM (2 mL) for 36-72 h prior to the day of the experiment and maintained at 37 °C in a 5% CO_2 atmosphere until they reached 80–90% confluence. Prior to flow cytometry, the hepatocytes under various treatments in each well were washed with PBS and trypsinized with 500 μL of 0.05% trypsin for 1 min. The trypsin was discarded, and 1 mL of high-glucose DMEM was added to each well. The cells were then centrifuged at 1000 rpm for 5 min, and the medium was discarded. The cells were resuspended in 1.5 mL of PBS and again centrifuged at 1000 rpm for 5 min. Next, each pellet was incubated with 195 μL of Annexin V-FITC binding buffer, 5 μL of Annexin V-FITC and 10 μL of propidium iodide (PI) at room temperature in the dark for 20 min. After incubation, the hepatocytes were centrifuged at 1000 rpm for 5 min and resuspended in 200 μL of PBS. After these procedures, the hepatocytes were subjected to flow cytometry. The fluorescence signals in the FITC channel and PI channel were measured for 5000 cells on an ImageStreamX Mark II flow cytometer (Merck). The data were analyzed using IDEAS software version 6.2.

Measurement of mitochondrial membrane potential

The change of mitochondrial membrane potential was quantitatively determined by flow cytometry using the Mitochondrial Membrane Potential Assay Kit with JC-1 (C2005, Beyotime). The accumulation of JC-1 within mitochondria with high mitochondrial membrane potential leads to the formation of J-aggregates, emitting a red fluorescence at 590 nm. When the mitochondrial membrane potential decreases, JC-1 exists as a monomer and produces green fluorescence at 529 nm. Hepatocytes were processed and collected in a manner consistent with the flow cytometric method described above. Afterwards, each pellet was incubated with 250 μL 10 μg/ml JC-1 solution at 37 °C for 30 min. After incubation, the samples were washed twice with 500 μL PBS, and finally suspended with 200 μL PBS. After these procedures, the hepatocytes were subjected to flow cytometry. The fluorescence signals in the JC-1 monomers and JC-1 aggregates were measured for 5000 cells on an ImageStreamX Mark II flow cytometer (Merck). The data were analyzed using IDEAS software version 6.2.

Statistical analysis

All data are expressed as the mean ± S.D. The data under each condition were accumulated from at least three independent experiments. For each experiment, unless otherwise noted, n represents the number of individual biological replicates. For each biological replicate and for all *in vitro* and *ex vivo* studies, $n \geq 3$. The Student's t test was used for comparisons between two groups of experiments. Statistically significant P values are indicated in Figures and/or legends as ***$P < 0.001$, **$P < 0.01$.

Data availability

All relevant data that support the findings of this study are available from the corresponding author upon reasonable request.

Results and Discussion

Synthesis of MB-Gly

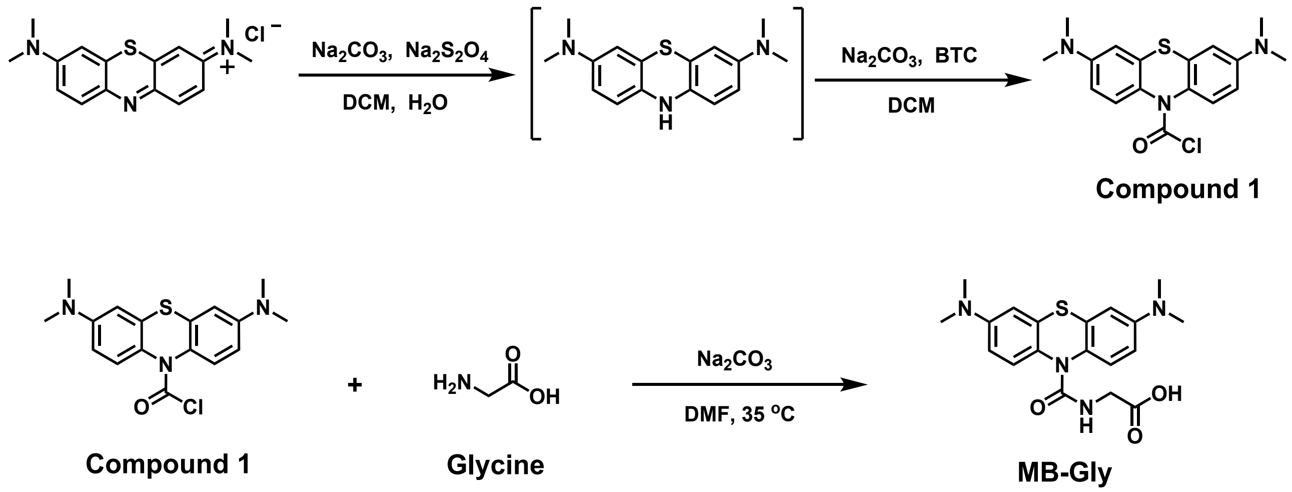

Scheme S1. The synthesis route of MB-Gly.

Synthesis of Compound 1

Compound 1 was prepared from methylene blue (MB) by one-pot reaction with leuco-MB (LMB) as the intermediate according to reported methods.[3, 4] Under a nitrogen atmosphere, methylene blue (1.60 g, 5.00 mmol) was added to dichloromethane (10 mL), followed by the addition of Na_2CO_3 (2.12 g, 20.00 mmol) in 20 mL of water. The mixture was stirred at 40 °C. $Na_2S_2O_4$ (3.48 g, 20.00 mmol) in 20 mL of water was injected to the solution and stirred for 30 min until the solution turned yellow. The mixture was cooled with an ice-water bath, to which bis(trichloromethyl)carbonate (0.89 g, 3.00 mmol) in 20 mL of dichloromethane was added dropwise. The solution was stirred at 0 °C for 3 h under a nitrogen atmosphere. Then, the solution was poured into 200 mL ice-water while stirring, and the resulting mixture was extracted with 3 × 100 mL portions of dichloromethane. The combined extracts were washed with brine, dried over anhydrous sodium sulfate and evaporated on a rotary evaporator. The crude product was subsequently purified by column chromatography, eluting with ethyl acetate/n-hexane (5:1, v/v) to afford compound 1 as a white solid (0.65 g,

1.87 mmol, 37%).

Synthesis of MB-Gly

Compound 1 (0.35 g, 1.00 mmol), Na_2CO_3 (0.42 g, 4.00 mmol) were dissolved in extra dry *N*,*N*-dimethylformamide (15 mL), followed by the addition of glycine (0.11 g, 1.5 mmol). Then the entire mixture was stirred at 35 °C for 6 h under a nitrogen atmosphere. The solutions were evaporated on a rotary evaporator, then the crude was subsequently purified by thin layer chromatography (dichloromethane/methanol = 5:1, v/v) to afford MB-Gly as a bluish white solid (0.12 g, 0.31 mmol, 31%). HRMS (ESI): calcd for $C_{19}H_{21}N_4O_3S$ [M - H]$^-$: 385.1339; found: 385.1323. ^{1}H NMR (400 MHz, DMSO-d_6): δ 7.29 (d, J = 8 Hz, 2H), 6.71-6.64 (m, 4H), 5.98 (t, J = 4 Hz, 1H), 3.54-3.45 (m, 2H), 2.88 (s, 12H). ^{13}C NMR (100 MHz, DMSO-d_6): δ 172.69, 154.89, 148.94, 133.53, 128.88, 127.81, 111.51, 110.73, 45.76, 40.69.

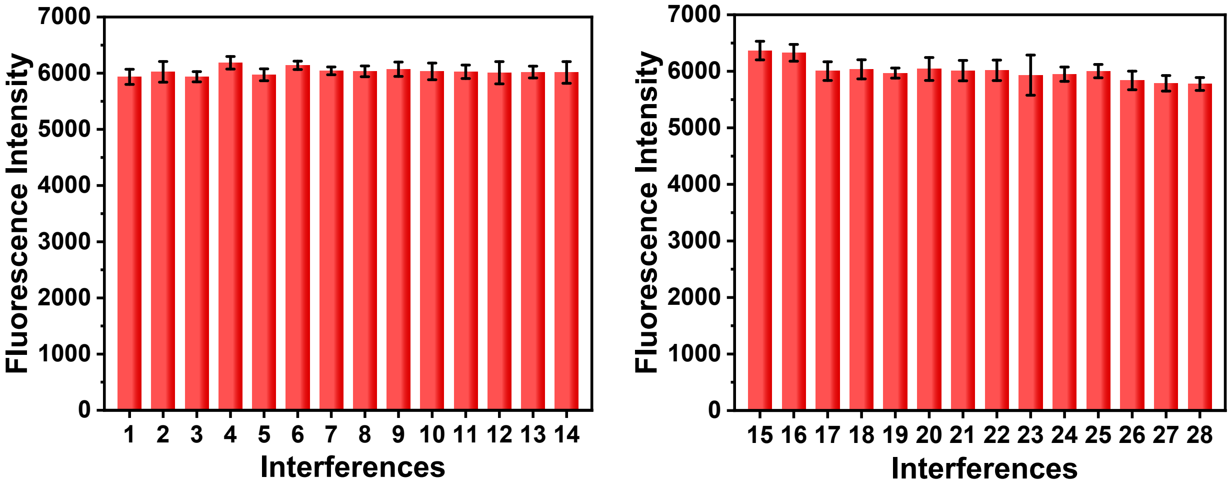

Figure S1. Fluorescence response at 687 nm of a mixture of MB-Gly (25 μM) and HOCl (38 μM) to various ROS, RNS, amino acids and metal ions (1-28: 10 mM H_2O_2, 100 μM $^1O_2$, 100 μM TBHP, 100 μM $^{\bullet}OH$, 100 μM $O_2^{\bullet-}$, 50 μM $NO^{\bullet}$, 50 μM $ONOO^-$, Blank, 10 mM K^+, 10 mM Na^+, 200 μM Ca^{2+}, 200 μM Mg^{2+}, 200 μM Zn^{2+}, 200 μM Fe^{2+}, 200 μM Fe^{3+}, 200 μM Cu^{2+}, 1 mM Pro, 1 mM Ser, 1mM His, 1 mM Lys, 1 mM Arg, 1 mM Asn, 1 mM Gln, 1mM Gly, 1 mM Asp, 1 mM Met, 1 mM Cys, 1 mM Hcy) in PBS buffer solution (10 mM, pH = 7.40). $\lambda_{ex/em}$ = 620/687 nm.

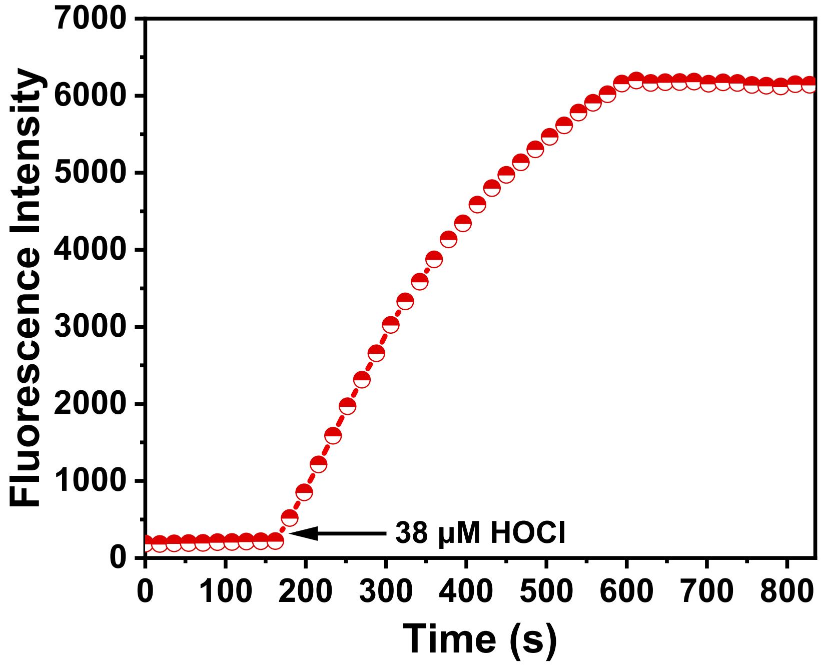

Figure S2. Emission at 687 nm of MB-Gly (25 μM) upon the addition of HOCl (38 μM) in PBS buffer solution (10 mM, pH = 7.40). $\lambda_{ex/em}$ = 620/687 nm.

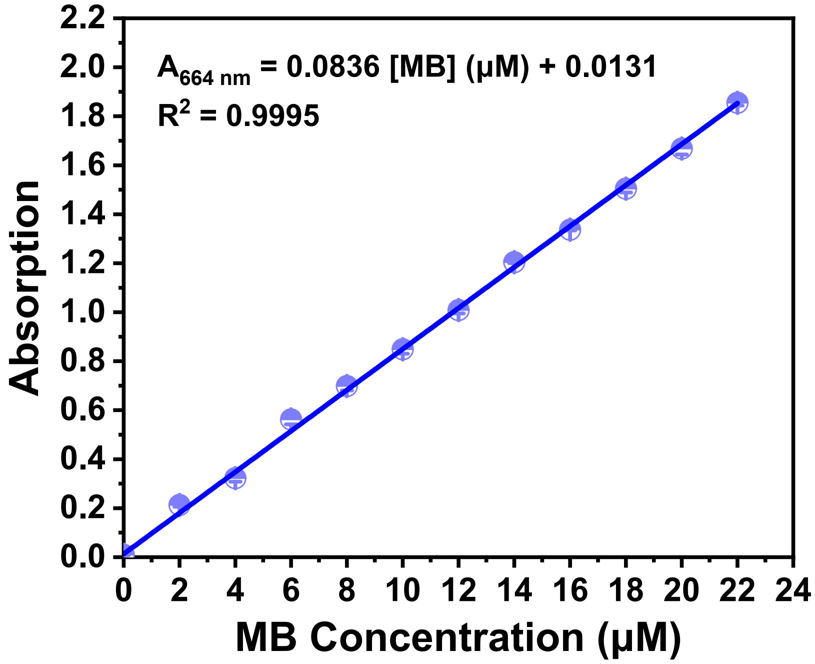

Figure S3. Calibration curve of absorbance intensity at 664 nm versus the concentration of MB.

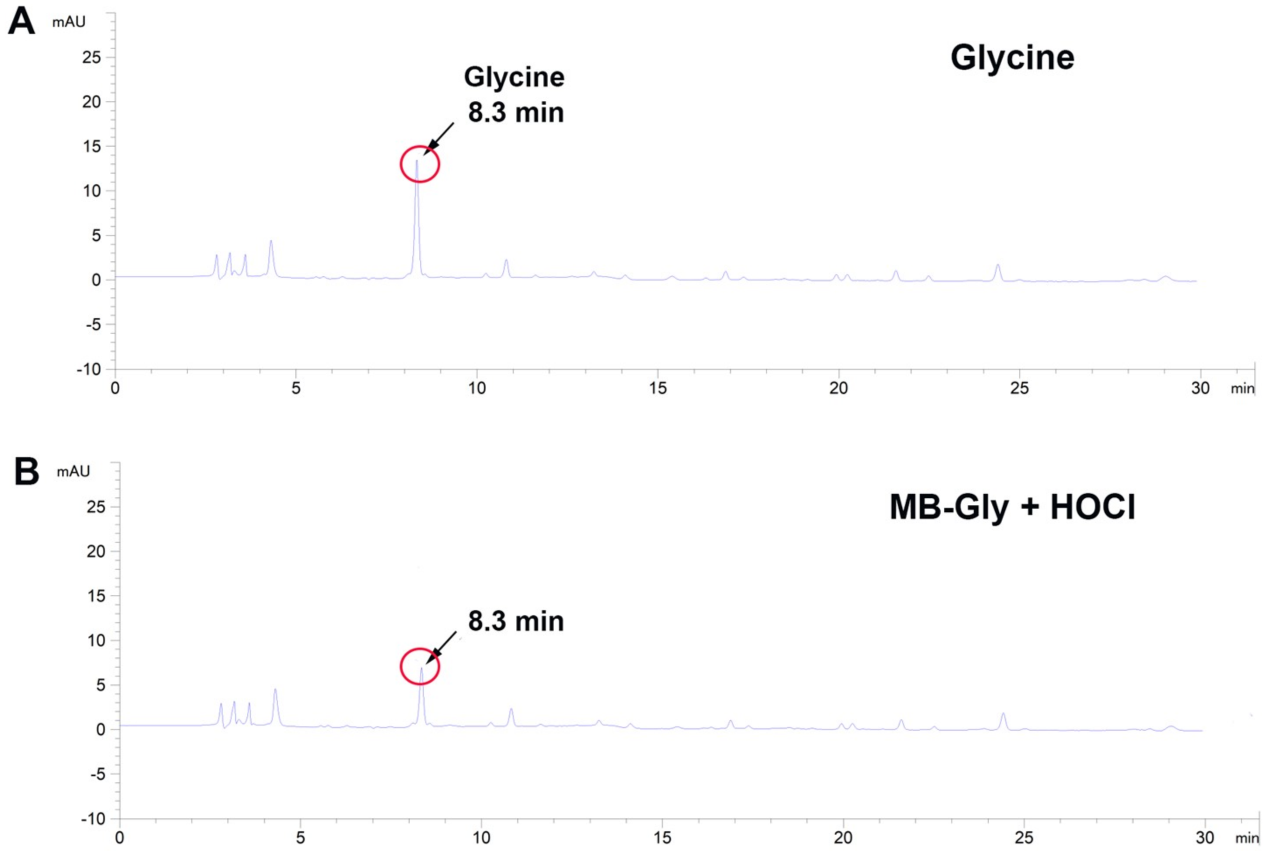

Figure S4. Analytical HPLC analysis of amino acid. (A) Glycine samples. (B) MB-Gly after reaction with HOCl. The retention time of 8.3 min represents glycine.

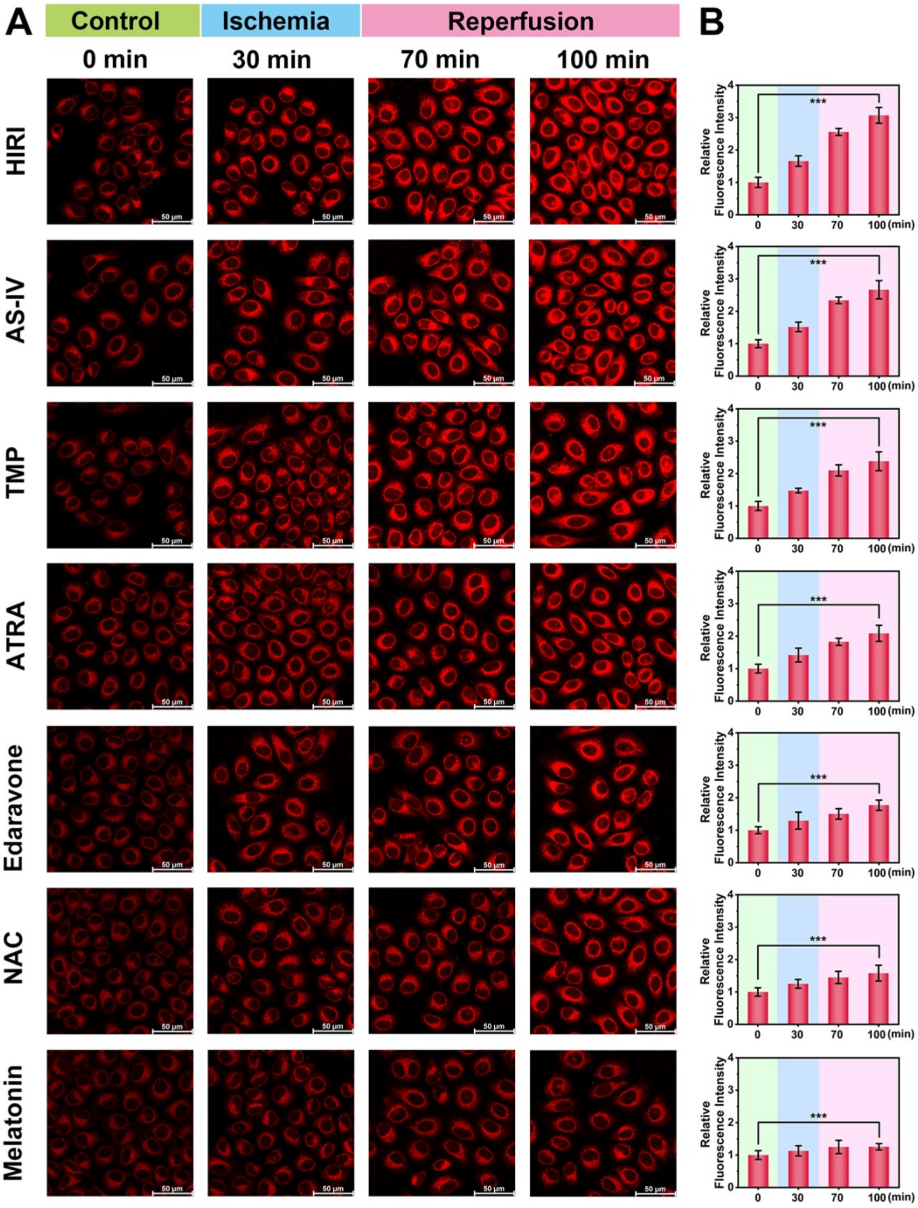

Figure S5. Dynamic fluorescence images of HIRI group hepatocytes, and HIRI group pretreated with As-IV (500 μM), TMP (500 μM), ATRA (500 μM), edaravone (500 μM), NAC (500 μM), melatonin (500 μM) by MB-Gly (50 μM, λ_{ex} = 638 nm, λ_{em} = 643-775 nm) during HIRI processes. The data are expressed as the mean ± SD. ***P < 0.001.

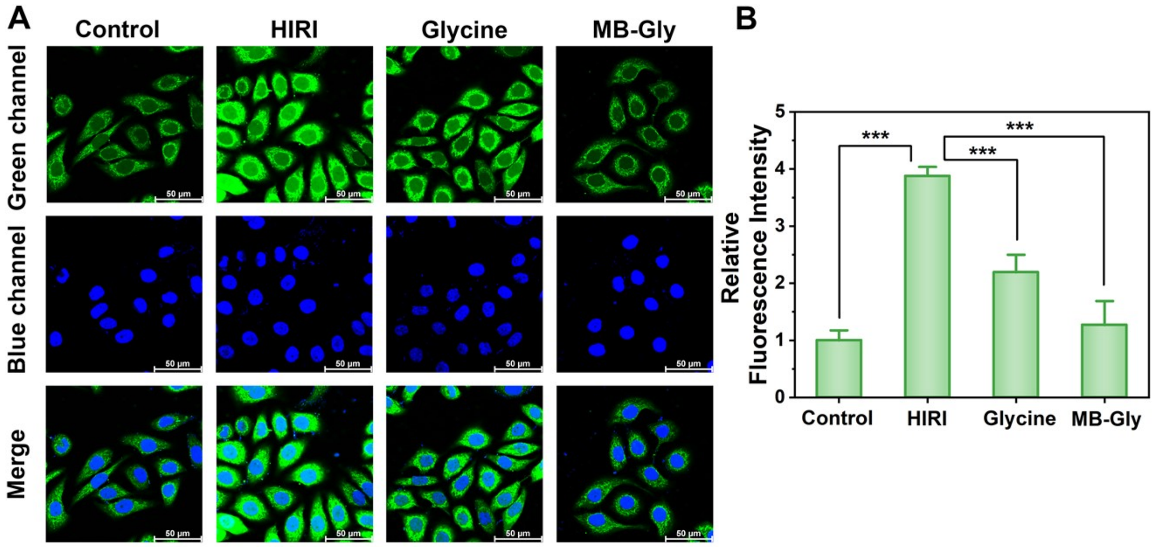

Figure S6. Confocal fluorescence imaging of ROS levels in HIRI hepatocytes under the treatment of glycine or MB-Gly. (A) Fluorescence imaging of ROS (green channel, λ_{ex} = 488 nm, λ_{em} = 495-580 nm) and nucleus (blue channel, λ_{ex} = 405 nm, λ_{em} = 420-480 nm) by DCFH-DA (10 μM) and Hoechst 33342 (1 μg/mL) in control group, HIRI group, glycine-treated HIRI group and MB-Gly-treated HIRI group hepatocytes. (B) Relative green fluorescence intensity output of (A). Note: The green fluorescence intensity of the control group was defined as 1.0. The data are expressed as the mean ± SD. ***$P < 0.001$.

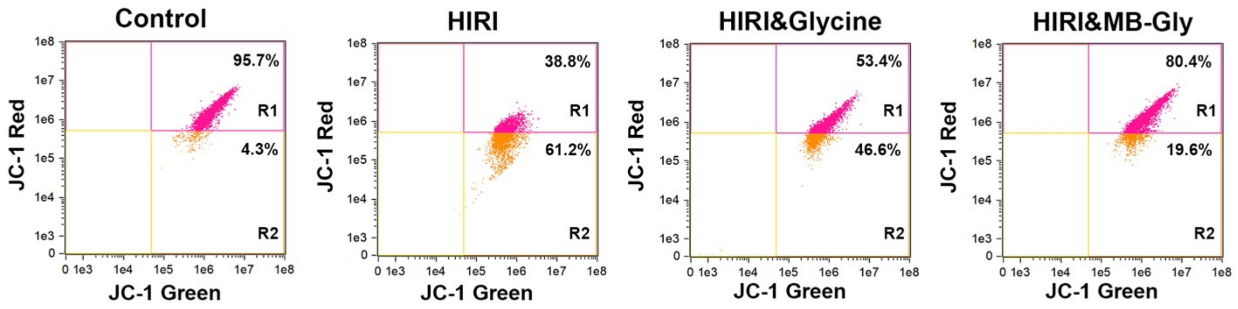

Figure S7. Mitochondrial membrane potential of hepatocytes in control group, HIRI group, glycine-treated HIRI group and MB-Gly-treated HIRI group. R1 region represents the proportion of hepatocytes with high mitochondrial membrane potential. When the mitochondrial membrane potential is high, JC-1 accumulates in the mitochondrial matrix and forms aggregates, producing red fluorescence. R2 region represents the proportion of hepatocytes with low mitochondrial membrane potential. In the cells with low mitochondrial membrane potential, the red fluorescence transforms into green fluorescence, suggesting the monomeric state of JC-1. The % values represent the proportion of hepatocytes with high mitochondrial membrane potential (R1 region) and low mitochondrial membrane potential (R2 region).

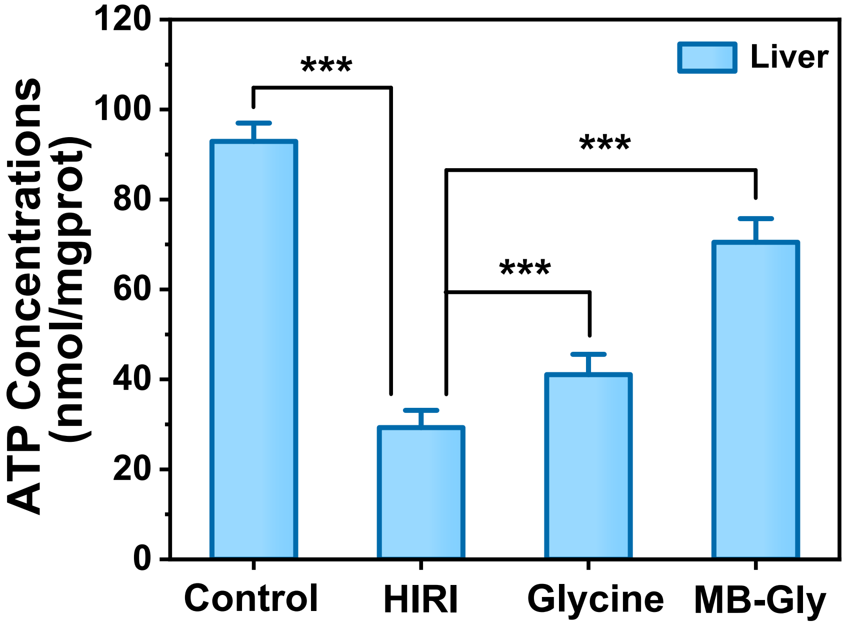

Figure S8. ATP content in liver tissues of mice in the control group, HIRI group, glycine-treated HIRI group and MB-Gly-treated HIRI group.

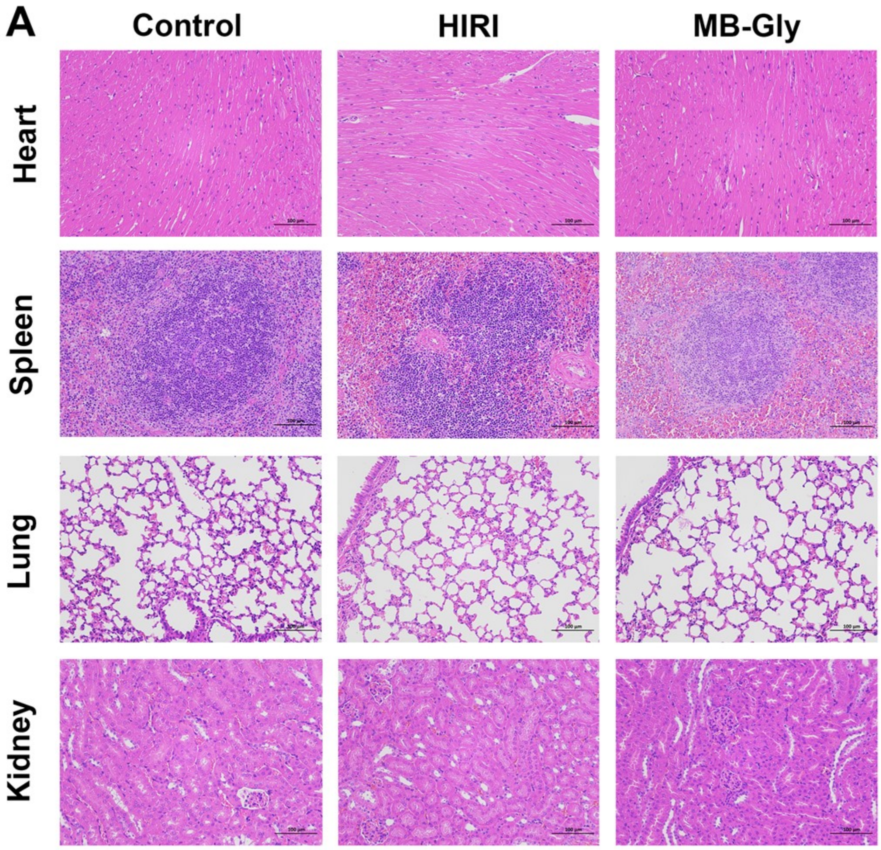

Figure S9. Hematoxylin and eosin (H&E) staining of the major organ tissues (heart, spleen, lung, and kidney) in the control group, HIRI group and MB-Gly-treated HIRI group.

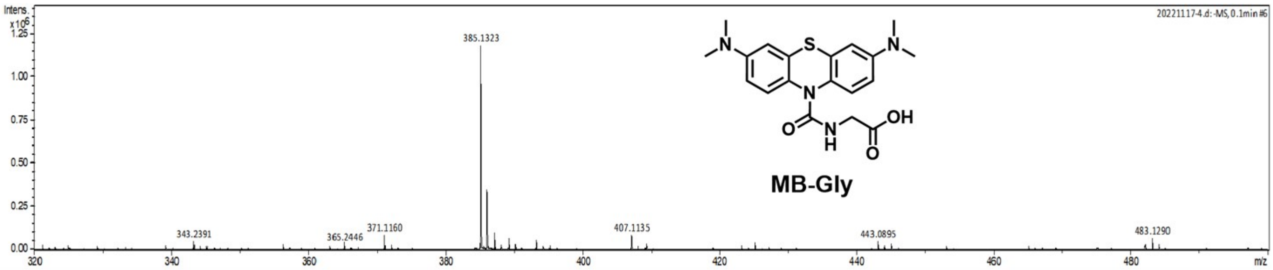

Figure S10. HRMS of MB-Gly.

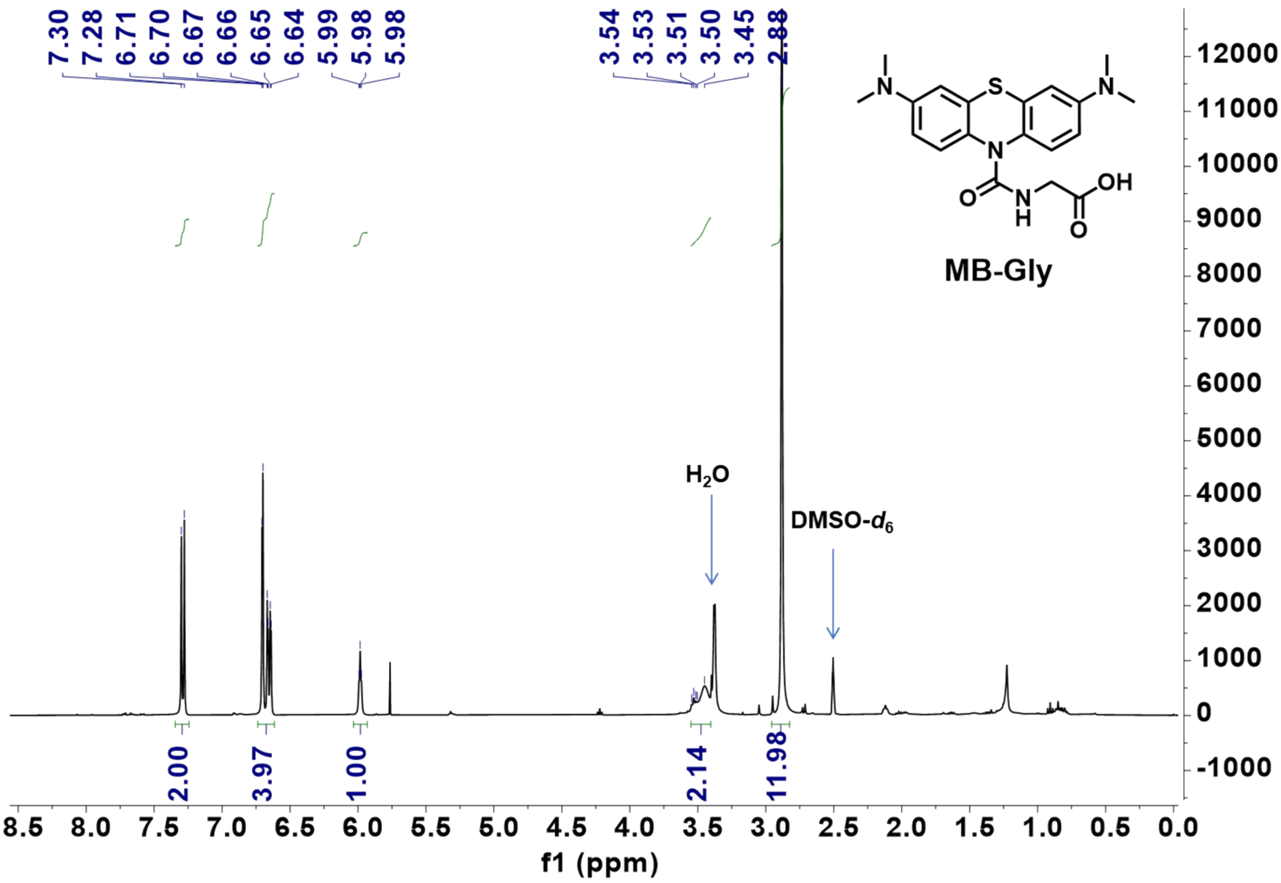

Figure S11. ^{1}H NMR (400 MHz, DMSO-d_6) of MB-Gly.

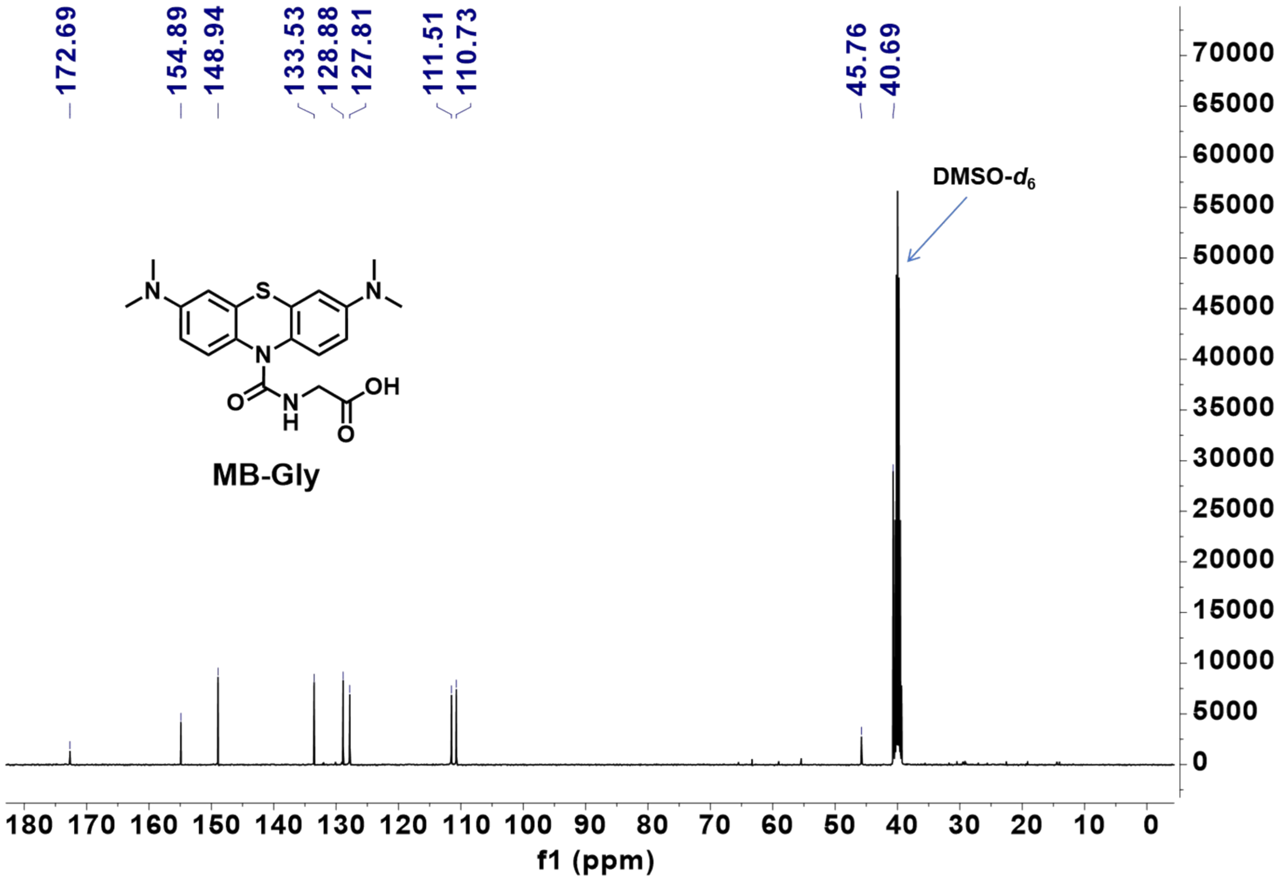

Figure S12. ^{13}C NMR (100 MHz, DMSO-d_6) of MB-Gly.

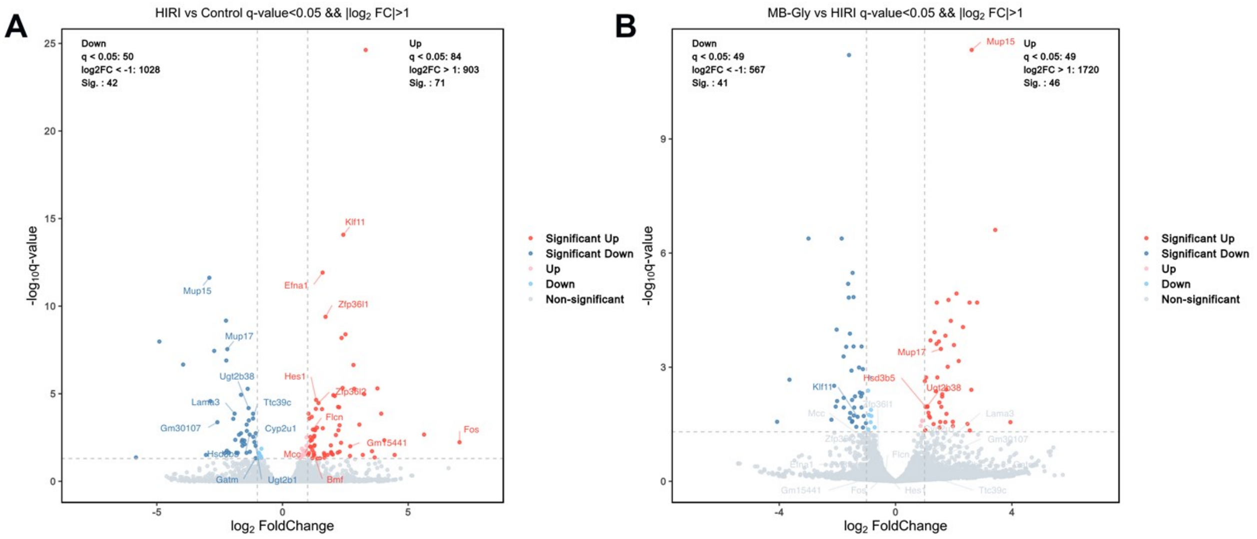

Figure S13. Volcano plots showing results of the DEGs in the HIRI group compared with control group (A) and the DEGs in MB-Gly-treated HIRI group compared with HIRI group (B). DEGs were identified using q value < 0.05 and $|\log_2 FC| > 1$ as the threshold parameters. Down-regulated DEGs are presented as blue dots while up-regulated ones are shown as red dots.

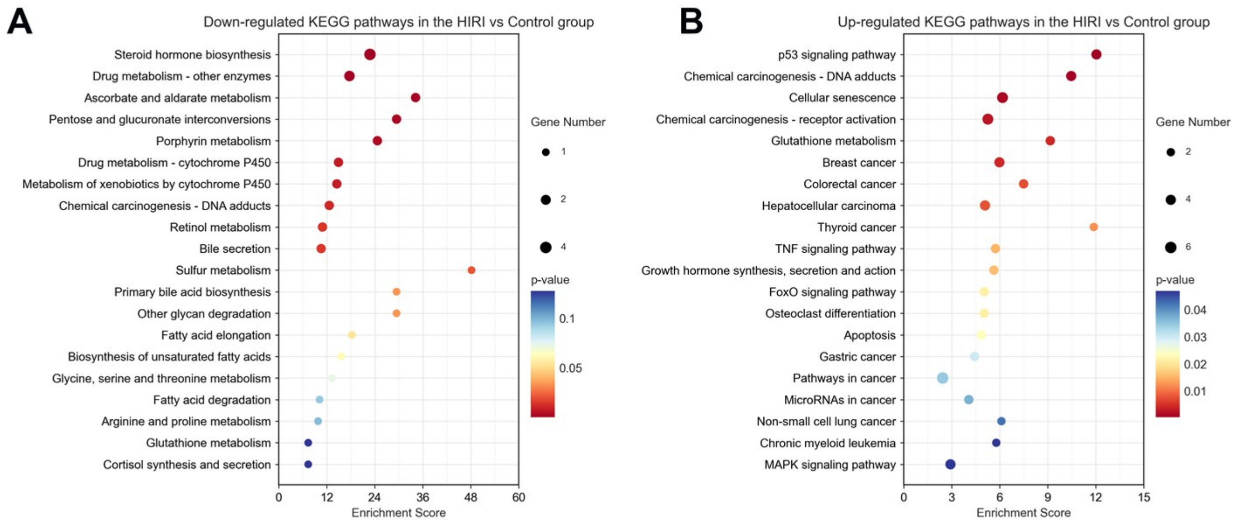

Figure S14. KEGG pathways of down-regulated (A) and up-regulated (B) genes in the HIRI group compared with the control group.

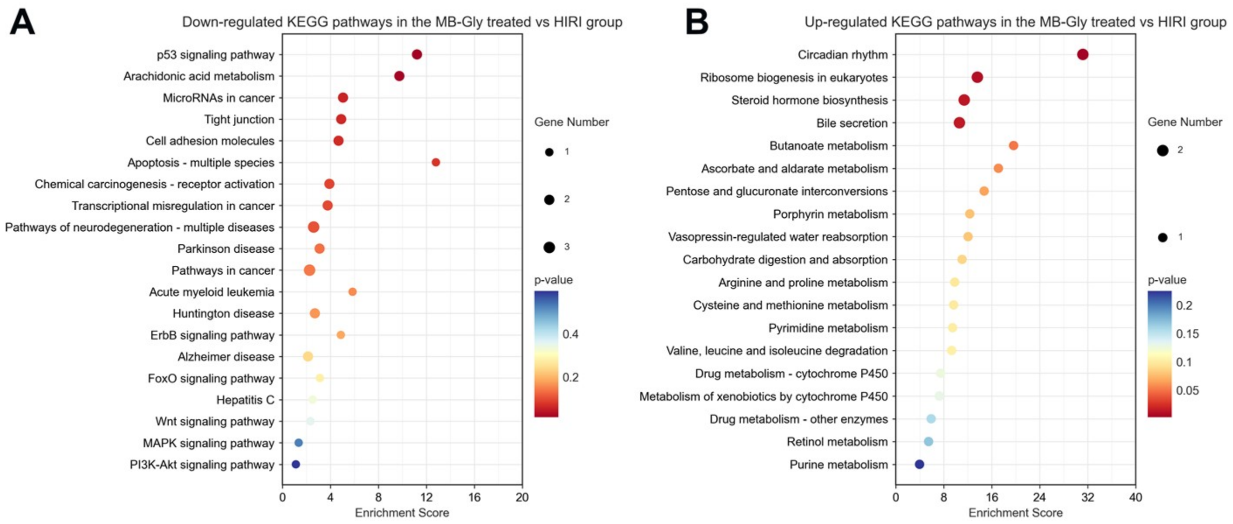

Figure S15. KEGG pathways of down-regulated (A) and up-regulated (B) genes in the MB-Gly-treated HIRI group compared with HIRI group.

References

1. X. Wang, P. Li, Q. Ding, C. Wu, W. Zhang and B. Tang, Illuminating the Function of the Hydroxyl Radical in the Brains of Mice with Depression Phenotypes by Two-Photon Fluorescence Imaging, *Angew. Chem. Int. Ed.*, 2019, **58**, 4674-4678.
2. J. Liu, W. Zhang, X. Wang, Q. Ding, C. Wu, W. Zhang, L. Wu, T. D. James, P. Li and B. Tang, Unveiling the Crucial Roles of $O_2^{\bullet-}$ and ATP in Hepatic Ischemia–Reperfusion Injury Using Dual-Color/Reversible Fluorescence Imaging, *J. Am. Chem. Soc.*, 2023, **145**, 19662-19675.
3. P. Wei, W. Yuan, F. Xue, W. Zhou, R. Li, D. Zhang and T. Yi, Deformylation reaction-based probe for in vivo imaging of HOCl, *Chem. Sci.*, 2018, **9**, 495-501.
4. Y. Tong, M. Li, H. Huang, S. Long, W. Sun, J. Du, J. Fan, L. Wang, B. Liu and X. Peng, Urea-Bond Scission Induced by Therapeutic Ultrasound for Biofunctional Molecule Release, *J. Am. Chem. Soc.*, 2022, **144**, 16799-16807.
